# Insight into the corrosion mitigation performance of three novel benzimidazole derivatives of amino acids for carbon steel (X56) in 1 M HCl solution

**DOI:** 10.1039/d3ra01837g

**Published:** 2023-04-27

**Authors:** Qahtan A. Yousif, Zainb Fadel, Ahmed M. Abuelela, Eid H. Alosaimi, Saad Melhi, Mahmoud A. Bedair

**Affiliations:** a University of Al-Qadisiyah, College of Engineering, Department of Materials Engineering Iraq qahtan.adnan@qu.edu.iq; b General Directorate of Education Al-Qadisiyah, Ministry of Education Iraq; c Department of Chemistry, Faculty of Science (Men's Campus), Al-Azhar University Nasr City 11884 Cairo Egypt; d Department of Chemistry, College of Science, University of Bisha P.O. Box 511 Bisha 61922 Saudi Arabia; e Department of Chemistry College of Science and Arts, University of Bisha P.O. Box 101 Al-Namas 61977 Saudi Arabia mbedair@ub.edu.sa m_bedier@yahoo.com

## Abstract

Three new organic molecules having a benzimidazole base were synthesized and used for the protection of carbon steel (X56) against corrosion in 1.00 M HCl solution. The protection against corrosion was assessed by electrochemical frequency modulation (EFM), electrochemical impedance spectroscopy (EIS) and potentiodynamic polarization (PDP). In addition, the electronic and molecular structure of the synthesized molecules were computationally investigated and correlated to corrosion inhibition. Global reactivity descriptors, molecular orbitals (FMO and NBO) and local reactivity descriptors (molecular electrostatic potential map and Fukui functions) were discussed. The results showed a maximum protective efficiency range between 95% and 98% indicating high corrosion inhibition. Moreover, all molecules were able to combat the cathodic as well as anodic reaction simultaneously, revealing a mixed-type resistance. SEM and EDX verified effective adhering film formation to the metal surface. In accordance, the theoretical calculations showed effective electron reallocation from the organic film to the X56 c-steel surface. Furthermore, the adsorption annealing calculations revealed that structural layers of these molecules hold parallel and close to the metal surface with adsorption energy from 249.383 to 380.794 kcal mol^−1^, showing strong inhibitor-metal contact.

## Abstract 2

Three organic molecules having benzimidazole were synthesized and used for protection of carbon steel (X56) from corrosion in 1.00 M HCl. They showed a maximum protective efficiency range between 95% and 98% indicating high corrosion inhibition.

## Graphical abstract

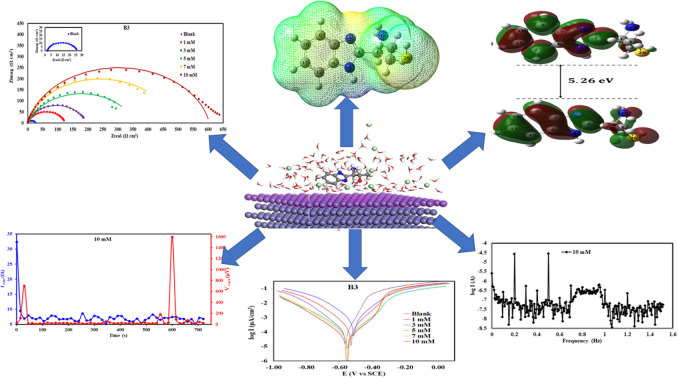

## 1. Introduction

Due to increased demand for useful fuels, upgrading heavy hydrocarbons to light fuels like diesel and gasoline has become more popular.^1^ The most difficult problem in the petroleum refining industry is producing high-quality fuels.^2,3^ Carbon steel pipelines, tanks, and the refinery's infrastructure that carry crude oil^4–6^ suffer from corrosion, which is a serious issue in the oil and gas industry, and it frequently results in equipment failure and distortion.^7,8^ Corrosion can occur when metals interact with crude oil elements like sulphur and naphthenic organic acids such as naphthalene and naphthene acids.^9–11^ Oil well acidification can also results in corrosion.^12,13^ Further research is needed to understand how these materials respond to corrosive conditions.^14^ Carbon steel (CS) has been included in considerable use in various situations in the petroleum industry.^15^ Among the most corrosive media, acid solutions, in water, are commonly employed in industry for pickling and acid cleaning of boilers and oil wells.^16–18^ Carbon-corrosion steel's resistance is significantly lower than other metals in acidic conditions^19^ and raised using a variety of approaches^20,21^ in which organic inhibitors were commonly used.^22^ The hydrochloric acid solution is commonly used in industrial applications such as cleaning, pickling, and galvanizing metals and it is also an important component of several industrial processes such as descaling, refining, and preparation.^23–25^ HCl is typically used at a concentration of 5–10% for this purpose, which results in a high rate of corrosive pitting and damage to the surface of steel construction components.^4,26,27^ Corrosion inhibitors are typically added to the acid wash solution to prevent corrosion.^28,29^

Almost all corrosion inhibitors are prepared from organic compounds with heteroatoms in their aromatic or cyclic structures.^30^ These compounds have a lot of heteroatoms (like N, S, P, and O) and many bonds, which makes it easier for the inhibitor to stick to the surface of the metal.^31,32^ Some quantum mechanical investigations have successfully related corrosion prevention efficacy and the molecular characteristics of certain organic molecules.^33–36^ Despite the huge number of organic compounds available, selecting an effective corrosion inhibitor for a specific system is limited by the specificity of the inhibitors and the wide range of corrosion systems.^19,20,37–39^ Scientists are still looking for corrosion inhibitors that are less expensive, non-toxic, and safe for both the environment and personnel. In furtherance of the search, the present work focuses on the synthesis of three novel water-soluble amino-acids benzimidazole derivatives (1-amino-1-(1*H*-bezo[*d*]imidazole-2-yl-2-ol, B1), 1-(1*H*-bezo[*d*]imidazole-2-yl)-3-methylbutan-1-amine, B2, and1-(1*H*-benzo[*d*]imidazole-2-yl)-3-(methylthio) propan-1-amine, B3) and investigation of their inhibition effect on the corrosion of carbon steel (Grade X56) in 1 M HCl solution using electrochemical methods in addition to SEM/EDX/mapping spectra studies. Moreover, a quantum chemical approach was used to relate the electronic properties of the synthesized molecules to the inhibitory effect on carbon steel.

## 2. Experimental part

### 2.1. Chemical materials and instrumentation

The threonine, leucine, and methionine amino acids and *ortho*-phenylenediamine employed in the present research are of analytical grade and were purchased from Sigma Aldrich; they were not further purified. Toluene solvent and hydrochloric acid were purchased from the Scharlau Company. Using matching silica cells and a Shimadzu UV-1800 spectrophotometer, the electronic spectra of the compounds were measured in an ethanol + water mixture (2 : 8). The KBr discs were used to record the infrared (IR) spectra of metal chelates across the range of 400 to 4000 cm^−1^ using a Fourier transforms infrared (FTIR) spectrophotometer (Shimadzu FTIR-8400S). Using D_2_O as a solvent, a Bruker DPX-300 spectrometer was used to record ^1^H and ^13^C NMR spectra with broadband decoupling of ^1^H at 600 MHz and 150 MHz. The compounds' elemental analyses (C, H, and N) were performed using the Flash EA 112 Elemental Analyzer. The percentage of the sample's constituents was determined using spectroscopy *via* emission and foundry-master Xpert instrument. After immersion in 1 M HCl solution with and without B1, B2, and B3 compounds, the surface morphology of carbon steel (Grade X56) coupons was analyzed using a field emission scanning electron microscope (FESEM) (ZEISS Sigma), energy-dispersive X-ray (EDX) spectroscopy, and mapping images.

### 2.2. Synthesis of benzimidazole derivatives

**Scheme 1:**
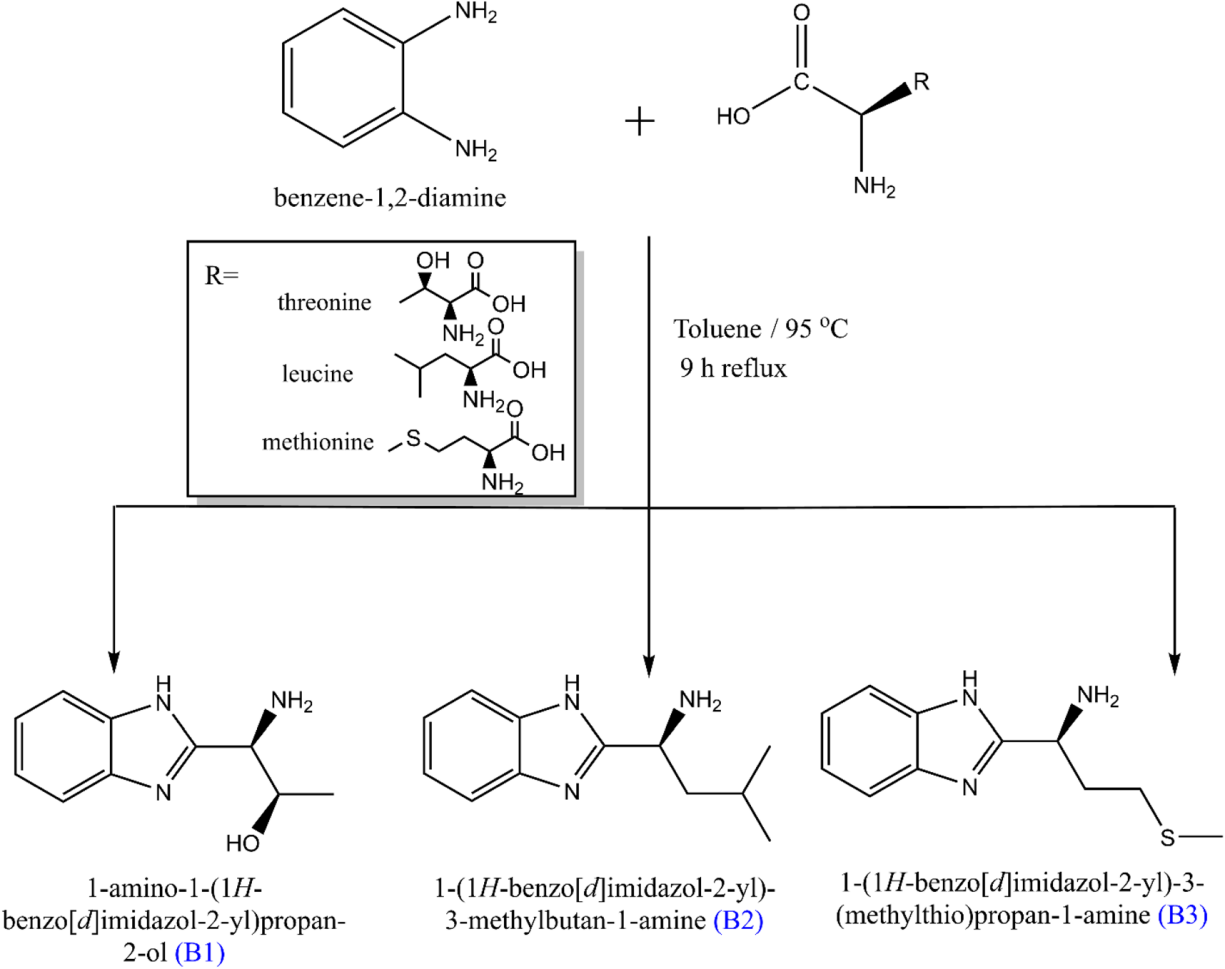
Synthetic route of the studied three amino acids derivatives.

O-phenylenediamine (5.0 mmol) was added to a round-bottom flask containing 10 mL of toluene and stirred for 40 seconds to ensure even miscibility. This solution was added dropwise to equimolar solutions of amino acids (threonine, leucine, and methionine, 5.0 mmol), and the color of the solution slightly changed. The mixture was heated under reflux for nine hours at a carefully controlled temperature of 85–95 °C.^40^ The solution was cooled and allowed to stand overnight to afford a colored solid, filtered by suction and oven-dried to afford the crystalline solid of the expected products B1, B2, and B3, as represented in Scheme 1. Thin-layer chromatography (TLC) was used to monitor the completion of the reaction.

### 2.3. Corrosion inhibition approach

The initial samples of carbon steel (Grade X56) were obtained from pipes transporting oil in southern Iraq. The required samples were then prepared in order to create the study's working electrodes. The element percentage of carbon steel alloy was determined utilizing emission manner at temperature of 24 °C and a humidity percentage of 32%. It was found to have the following percentages (wt%): carbon (0.174), manganese (1.36), silicon (0.367), sulfur (0.0035), phosphor (0.0116), nickel (0.0333), copper (0.0339), vanadium (0.0039), chromium (0.012), molybdenum (less than 0.002), and iron (balance). To prepare the working electrode, the mechanical method was used to cut the carbon steel into specimens of 1.5 cm × 1.5 cm × 1 cm dimensions. After that, they were encased in an epoxy resin, and the exposed area of 1 cm^2^ was abraded by hand using an angle grinder. This was done to remove any excess epoxy resin and then various grades of emery paper (600–1200) were used to remove rust and scale. Before the samples were used, the surface of each one was meticulously cleaned with distilled water, degreased with acetone and hot benzene, and then air-dried at room temperature. The potentiostat/galvanostat device was used for the electrochemical tests at 298.15 K. The Echem analyst program was used to gather and analyze the data. The carbon steel (Grade X56) was the working electrode in the electrochemical setup. The saturated calomel electrode (SCE) was the reference electrode while the platinum foil was the counter electrode. All three electrodes were immersed in the 1 M HCl solution. Prior to the experiments, the carbon steel sample was allowed to corrode naturally without any external current or potential for 1800 s to produce a stable open-circuit potential (OCP). The studies were conducted with and without B1, B2, and B3 inhibitors. Carbon steel samples were tested for electrochemical potentials using the PDP technique with the potential ranges from 0.4 to +0.6 at a scan rate of 0.2 mV s^−1^. The corrosion potential (*E*_corr_), cathodic and anodic Tafel slopes (*β*_c_ and *β*_a_), corrosion current density (*i*_corr_), and corrosion rate (*k*) were all extrapolated from Tafel plots. Using eqn (1) and (2) we can determine the inhibition efficiency (*η*_p_ (%))^41,42^ and corrosion rate.^43^1
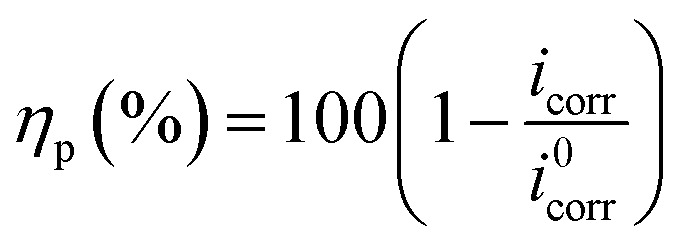
2
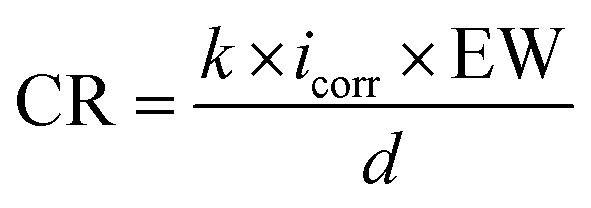


As, *k* is a conversion factor, *i*_corr_ is the corrosion current density, EW is the equivalent weight, and *d* is the density. The corrosion current densities in the absence and presence of different inhibitor concentrations are denoted by *i*^0^_corr_ and *i*_corr_, respectively. The impedance measurements were performed in the frequency response spectrum range of 1 Hz to 100 kHz at amplitude of ±10 mV. An equivalent circuit was utilized for fitting impedance spectrum data. Estimating parameters such as the charge transfer resistance*R*_ct_, solution resistance*R*_s_, and inhibition efficiency (*η*_*z*_ (%)) is essential. The impedance spectrum was represented using Nyquist and Bode plots. The corrosion inhibition efficiency, denoted by *η*_*z*_ (%), was determined by applying the following eqn (3) to the data:^44,45^3
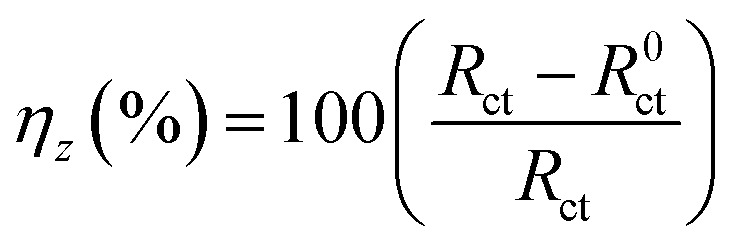
where *R*_ct_ and *R*^0^_ct_ represents the charge transfer resistances in the presence and absence of the studied inhibitor. In addition, electrochemical noise (EN) tests were performed. Two carbon steel samples were employed as working electrodes while a saturated calomel electrode (SCE) was used as a reference electrode. The electrochemical cell was placed in a Faraday cage to eliminate electrical interference. The exposed surface area of each working electrode was 1 cm^2^ and the distance between them was approximately 1 cm. The electrochemical current noise was measured using a galvanic coupling current between two working electrodes. The electrochemical potential noise was measured under free corrosion conditions between the reference electrode and two connected working electrodes. Current and potential noises were recorded for 720 seconds at a frequency of 5 Hz and a repeat time of 15 seconds. A low-pass filter with a frequency of 2.5 Hz was utilized to reduce frequencies over the Nyquist frequency during data recording. The following eqn (4);^46^ was used to derive the inhibitory performance values from the EN measurements:4
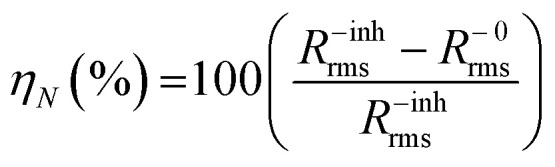
where *η*_*N*_ the percentage of inhibition efficiency, *R*^−0^_rms_ is the average noise resistance in an inhibitor-free medium, and *R*^−inh^_rms_ is the average noise resistance in an inhibitor-containing medium. In addition, the EFM criteria were satisfied by employing a potential perturbation signal (10 mV amplitude) with two sine waves at 2 and 5 Hz and 16 cycles. The selection of these frequencies was based on three justifications. The more prominent peaks were used to determine the corrosion current density (*i*_corr_), the Tafel slopes (*β*_c_ and *β*_a_) and the causality factors (CF-2 and CF-3). Eqn (5);^47,48^ can be used to determine efficiency as a function of current density.5
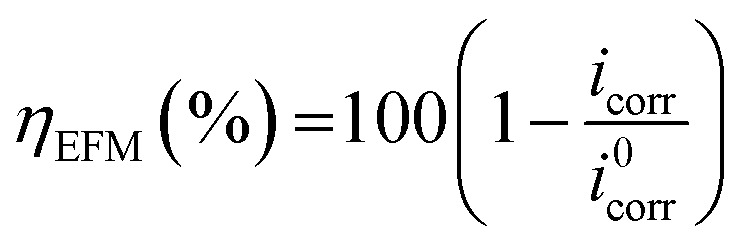


Electrochemical experiments were duplicated at each concentration of the tested inhibitors to ensure the experimental data's accuracy.

### 2.4. Computational investigation

Initial guesses of B1, B2 and B3 molecules were prepared by ChemBioDraw Ultra 14.0 in 2D dimensions then a minimization (energy/geometry) was performed by Gaussian 09 revision-A.02-SMP package^49^ at RHF/6-31g level. The optimized parameters were reached after 23 iterations by achieving the convergence criteria indicated by Gaussian 09: the maximum remaining force on an atom and the average (RMS, root mean square) force on all atoms are below the tolerance threshold 45 × 10^−5^ and 30 × 10^−5^ Hartrees, respectively as well as the maximum structural drift of one coordinate and RMS change over all structural parameters in the last two iterations are below 18 × 10^−4^ and 12 × 10^−4^ Å, respectively. It is worthy to mention that the maximum force and RMS force were two orders of magnitude smaller than the indicated thresholds (*i.e.*: 38 × 10^−6^ and 7 × 10^−6^ Hartrees, respectively) which reveal that a stationary point is reached regardless of the obtained values of the displacements. The obtained optimized parameters were used to re-optimize the structures by Density Functional Theory at B3LYP/6-31g(d,p) level of theory^50–52^ as well as carrying out Hirshfeld population analysis. Similar convergence criteria to that of RHF calculations were obtained after 42 iterations though. Obtained molecules representing local minima were sketched by Gaussview 6.^53^

Often DFT calculations underestimate Frontier molecular orbitals eigenvalues, therefore the DFT calculations were repeated using TD-DFT method to calculate the lowest excited state energy and therefore correct the value of Δ*E*_gap_ between HOMO and LUMO. Six energy states were included for this calculation at TD-B3LYP/6-31g(d,p) following the procedure described by ref. 54. *E*_HOMO_ and *E*_LUMO_ as well as their corresponding reactivity descriptors were obtained as described by ref. 54. HOMO and LUMO surfaces with isovalue of 0.02 and density of 0.0004 were presented using the molecular orbital editor of Gauss view 6.^53^ Natural bond orbitals (NBOs) were calculated at B3LYP/6-31g(d,p) level and presented for selected sites of B1, B2 and B3 molecules. NBOs surfaces were obtained using the NBO Version 3.1 (ref. 55) in Gaussian 09 software and plotted using the same isovalue of 0.02 and density of 0.0004 in order to compare them to FMOs. In addition, Fukui functions were calculated by adding a positive and a negative charge to the neutral molecules and then re-optimizing it at B3LYP/6-31g(d,p). The calculated condensed Fukui functions *f*_*k*_^+^ and *f*_*k*_^−^ were extracted.

A full view of the corrosion system (steel/corrosive medium/inhibitor) was considered using the adsorption locator tool in materials studio software.^56^ Two forms of the inhibitor's molecules (neutral and protonated) were used beside the addition of water molecules (200 H_2_O), hydronium ions (19 or 20 H_3_O^+^) and chloride ions (20 Cl^−^). The Fe (1 1 0) crystal [(11 × 11) super cell, 30 Å vacuum thickness and a dimension of 22.341 × 22.341 × 48.422 Å] was chosen as the X56 c-steel surface due to its compact structure and relatively low energy. The Monte Carlo annealing simulations were restricted to the criteria of COMPASS for force field interactions, group based for the electrostatic interactions and atom-based summation methods for van Der Waals interactions.

## 3. Results and discussion

### 3.1. Characterization of the inhibitors

#### 3.1.1. The physicochemical properties

The physicochemical properties of the B1, B2, and B3, such as color, melting point, and elemental percentages, have been determined. The resulting B1 compound is a solid, light brown powder. The yield was 94% with a melting point of 211–213 °C. Both yield and melting point values for the B2 and B3 were 90%/212–214 °C and 97%/205–207 °C, respectively. The elemental analyses were exhibited according to N%, C%, and H% (18.69, 49.73, and 4.613), (15.31, 43.17, and 3.789), and (13.03, 52.13, 4.701, and 11.15 (S%)) for B1, B2, and B3 compounds, respectively.

#### 3.1.2. FTIR analysis

**Fig. 1:**
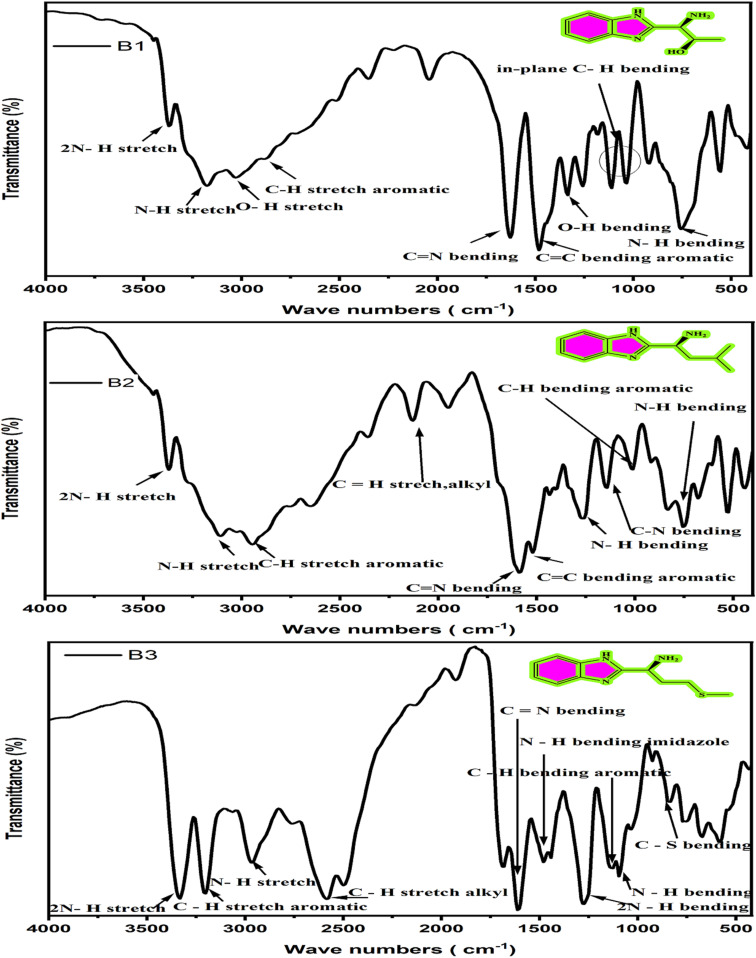
FT-IR spectra of the amino acids derivatives (B1, B2 and B3).

It is worthwhile that the chemical structures of B1, B2, and B3 were highlighted by FT-IR, as illustrated in Fig. 1.The FTIR spectrum (ν cm^−1^) of B1 shows different absorption bands that confirm its functional groups such as at 3386 (m, 2N–H), 3282 (m, N–H), 3175 (b, O–H), 3030 (m, C–H aromatic), 1650 (m, C=N), 1600 (m, C=C aromatic), 1419 (m, O–H), and 1593 (m, N–H). Confidently, the FT-IR spectrum of B2 displays various absorption bands verifying its functional groups like at 3279 (s, N–H), 2954 (s, C–H aromatic), 3386 (m, 2N–H), 2360 (m, C–H alkyl), 1696 (m, C=N), 1519 (m, C=C aromatic), 1149 (s, C–N), 1265 (656), 656 (m, N–H), and 1018 (s, C–H aromatic). The FT-IR spectrum of B3 shows many absorption bands each corresponding to a different functional group. Some of these bands are 3209 (s, C–H aromatic), 3332 (m, 2N–H), 2584 (m, C–H alkyl), 1681 (m, C=N), 848 (m, C–S), 1026 (m, C–H), 671 (m, N–H), 1473 (s, N–H imidazole ring), and 1272 (m, 2N–H).

#### 3.1.3. ^1^H and ^13^C NMR analysis

**Fig. 2:**
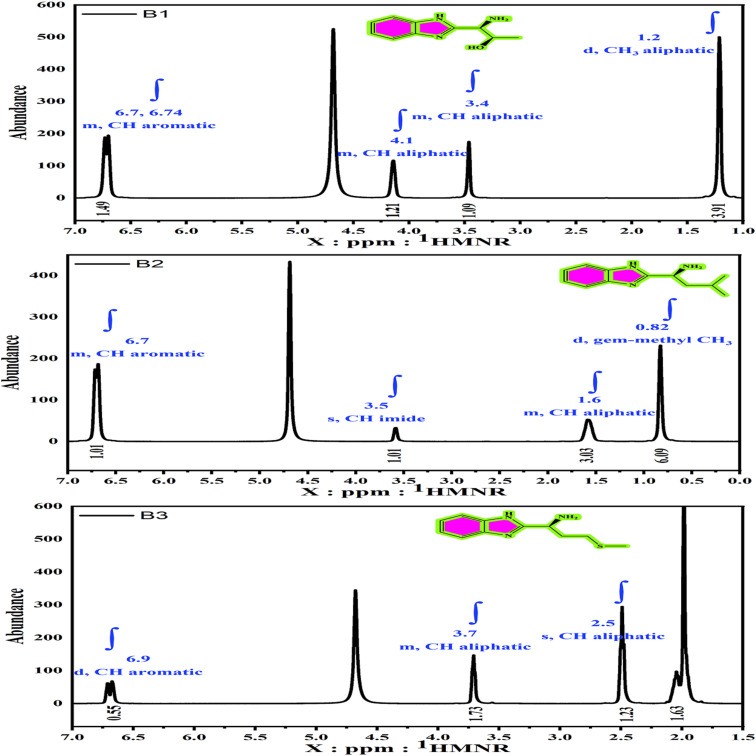
^1^H NMR spectra of the amino acids derivatives (B1, B2 and B3).

**Fig. 3:**
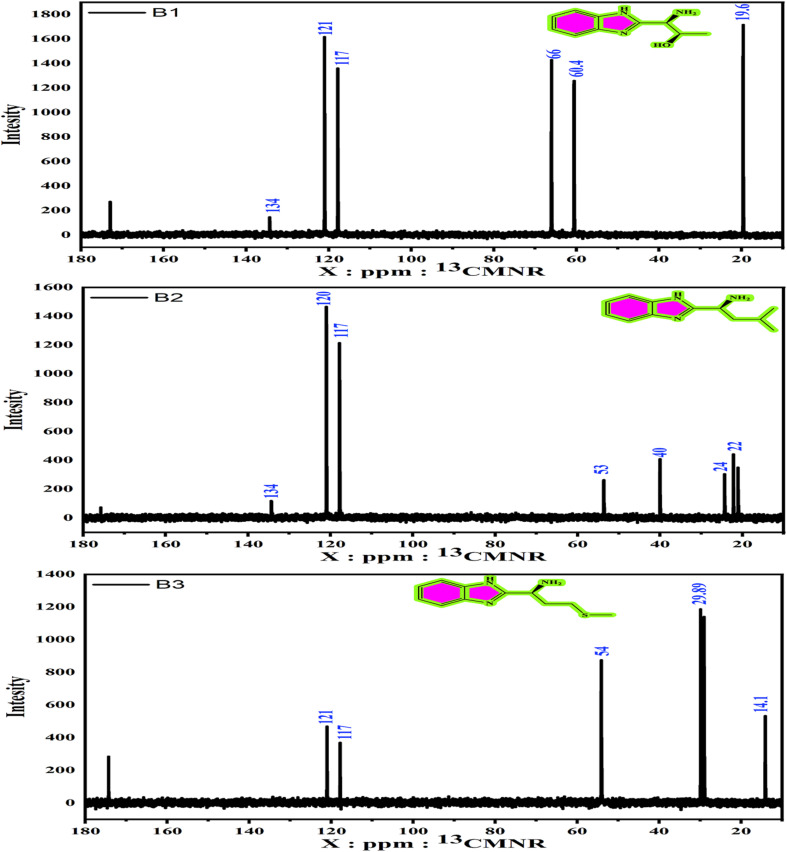
^13^CNMR spectra of the amino acids derivatives (B1, B2 and B3).

The ^1^H and ^13^C NMR spectra are shown in Fig. 2 and 3. The ^1^H NMR (D_2_O) of B1, B2, and B3 elucidated different signals (Fig. 2) at: *δ* (ppm) = 3.4 (m, C-H1 aliphatic), 1.2 (d, C-H3 aliphatic), 4.1 (m, C-H2 aliphatic), 6.70 (m, C-H11, 12 aromatic), and 6.74 (m, C-H13, 10 aromatic) for B1. The *δ* (ppm) values for the B2 are 0.82 (d, gem-methyl CH3–14, 15), 1.6 (m, C-H12, 13 aliphatic), 3.5 (s, C-H11 imide), and 6.7 (m, C-H4, 5, 6, 7 aromatic). Also, the singles for B3 are *δ* (ppm) = 1.9 (s, C-H2, 3 aliphatic), 3.7 (m, C-H1 aliphatic), 2 (m, C-H6), 2.5 (C-H3 aliphatic), and 6.6 (d, C-H12, 15, 13, 14 aromatic). The difference in signals is caused by variations in molecular configuration. Moreover, the ^13^C NMR (D_2_O) of B1, B2, and B3 ascribed different signals, as shown in Fig. 3 at *δ* (ppm) = 121 (C13, C14), 134 (C7, C8), 117 (C10, C13), 60.4 (C1), 66 (C2), and 19.6 (C2)3 for B1, and 22 (C15, C14), 24 (C13), 40 (C12), 53 (C11), 117 (C4, C7), 120 (C5, C6), and 134 (C1, C2) for the B2 structure. The chemical shifts *δ* (ppm) = 54 (C1), 117 (C12C13), 121 (C13, C14, C7, C9, C10), 29.05 (C3), 29.89 (C2), and 14.1 (C6) for the B3.

#### 3.1.4. UV-visible analysis

**Fig. 4:**
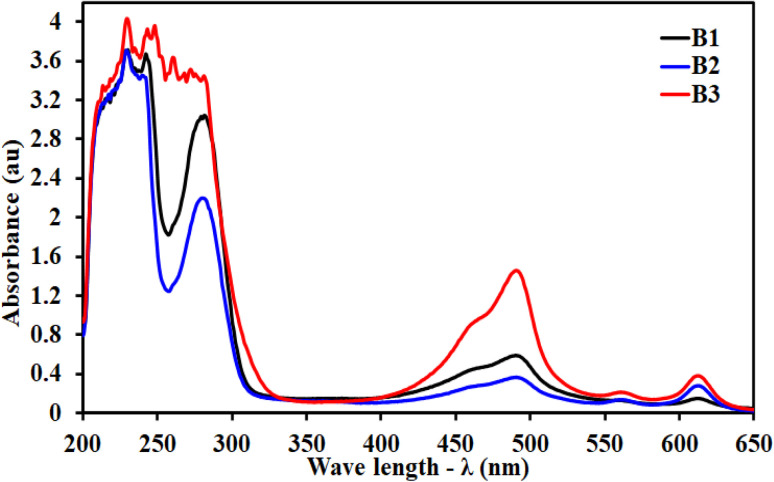
UV-visible spectra of the amino acids' derivatives (B1, B2 and B3).

The UV/visible spectra of B1, B2, and B3 were recorded in a range of 200–650 nm in H_2_O as a solvent at a concentration of 1 × 10^−4^ M at room temperature as shown in Fig. 4. An absorption band was observed between 228 and 267 nm which could be associated with a π–π* transition originating primarily in the azomethine chromophore and the n–π* transition electronic for the imidazole aromatic ring in 488 nm for the B1 structure. The results for B2 showed bands at 230 nm and 280 nm, both assigned to π–π* transitions. The band at 498 nm was assigned to n–π* transitions. The results for B3 were a strong band at 229 nm and a weak band at 272 nm. Both bands were attributed to π–π* transitions. The n–π* transitions in the imidazole aromatic ring were related to the band at 490 nm. When compared to the π–π* transition that observed in the two electronic bands, the absorption bands assigned to the n–π* transition have different values for the three compounds, which are related to the nature of the chromophore that responded to the kind of transition with respect to the nature of the solvent that was used.

### 3.2. Electrochemical measurements

#### 3.2.1. PDP measurements

**Fig. 5:**
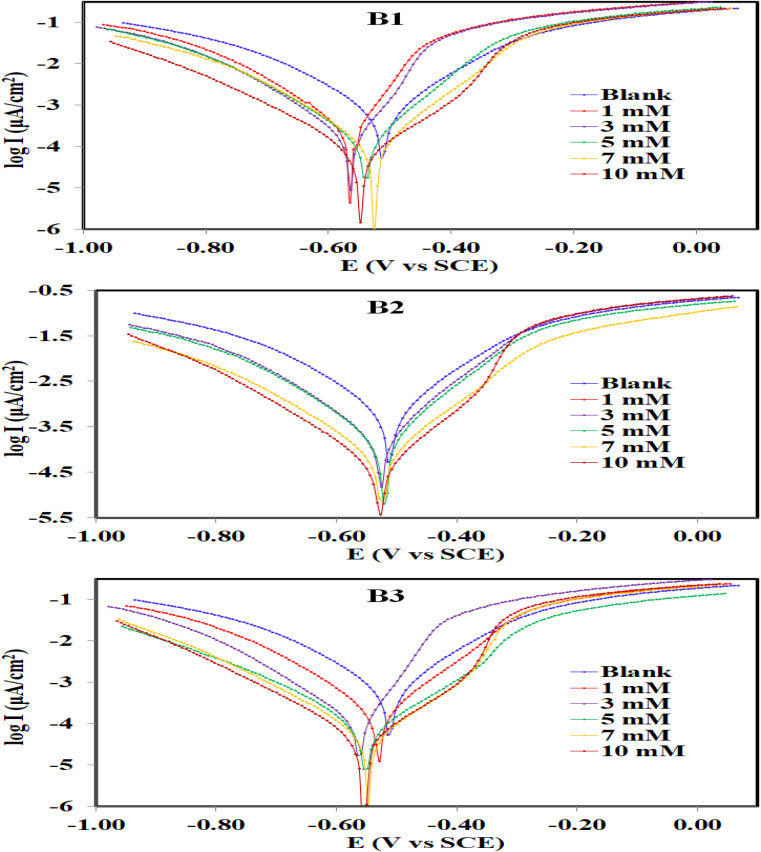
Potentiodynamic polarization curves for the corrosion of carbon steel in 1.0 M HCl in absence and presence of different concentrations of B1, B2 and B3 compounds at 30 °C.

Potentiodynamic polarization (PDP) was used to determine how fast the cathodic and anodic reactions were on the carbon steel surface. Based on the answer, it can be figured out how the inhibitors (B1, B2, and B3) affect the corrosion reaction of carbon steel in an aerated 1.0 M HCl solution at 30 °C. Fig. 5 depicts the curve of potentiodynamic polarization measurements (Tafel plots) for uninhibited and inhibited (with various concentrations) carbon steel corrosion. The main point noticed from the figure is that the addition of B1, B2 and B3 causes a displacement of both curves (anodic and cathodic) towards the lower side of *i*_corr_ when compared to the blank curve, which reveals towards the reduction in the corrosion reactions. Table 1 displays the inhibition efficiency and corrosion reaction data (corrosion potential (*E*_corr_), corrosion current density (*i*_corr_), anodic Tafel slope (*β*_a_), and cathodic Tafel slope (*β*_c_)) obtained by extrapolating the linear region of the polarization plot. Table 1 demonstrates that when B1, B2, and B3 inhibitors were added, the corrosion current density (*i*_corr_) values dropped dramatically compared to the uninhibited solution. Furthermore, as the inhibitor concentration increases, the *i*_corr_ values decrease and the inhibition efficiency (*η*_p_ (%)) increases.^57^

**Table 1:** Electrochemical parameters for carbon steel dissolution in 1.0 M HCl solution containing different concentrations of the (B1, B2 and B3) inhibitors obtained from polarization measurements at 30 °C^a^

Inhibitor name	Conc. (mM)	*E*_corr_ *vs.* SCE (mV)	*i*_corr_ (μA cm^−2^)	*β*_a_ (mV dec^−1^)	*β*_c_ (mV dec^−1^)	*k* (mpy)	*θ*	*η*_p_ (%)
Blank	—	−512	2300	391.9	250.9	1211	—	—
B1	1	−565	765	207.9	181.7	403.0	0.6674	66.74
	3	−565	455	190.3	173.0	239.7	0.8022	80.22
	5	−540	304	181.0	167.6	160.3	0.8678	86.78
	7	−526	207	176.7	166.2	108.8	0.9100	91.00
	10	−548	113	167.4	159.3	59.49	0.9509	95.09
B2	1	−522	703	324.3	315.3	321.3	0.6943	69.43
	3	−526	409	193.2	183.6	215.5	0.8222	82.22
	5	−521	267	188.2	172.1	140.9	0.8839	88.39
	7	−525	128	179.4	171.4	67.49	0.9443	94.43
	10	−528	72.3	154.4	151.7	38.07	0.9686	96.86
B3	1	−530	422	193.5	175.3	222.4	0.8165	81.65
	3	−563	260	175.8	161.3	136.9	0.8870	88.70
	5	−551	133	181.8	177.0	69.94	0.9422	94.22
	7	−547	78.6	157.2	154.1	41.41	0.9658	96.58
	10	−556	24.2	139.2	126	12.47	0.9895	98.95

a *E*_corr_, is the corrosion potential; *i*_corr_, is the corrosion current density: *β*_a_ and *β*_c_ are Tafel constants for both anode and cathode; *k*, is the corrosion rate; *θ*, is the surface coverage; *η*_p_, is the inhibition efficiency.

B3 seems to be a better inhibitor than B1 and B2. This means that it is more likely to slow down the anodic and cathodic reactions on carbon steel under the conditions of the experiment. This means that B3 is an efficient corrosion inhibitor. As the inhibitor is introduced, the molecules adsorb on the metal surface and obstruct the active sites. The highest *η*_p_ (%) of B3 is attained at the highest inhibitor concentration because the active sites are inhibited more severely at the higher inhibitor concentration which is 10 mM in this case. Fig. 5 shows that when B1, B2, and B3 inhibitors were present, both the anodic and cathodic currents were slowed down. This is because the Tafel regions in the polarization curves of the solution under study were well delineated, and the inhibiting action of these inhibitors on both cathodic and anodic processes is approximately the same. For that, the inhibitor may decrease the corrosion rate through the reduction of carbon steel reactivity and a decrease in the anodic reaction, cathodic reaction, or both that arise from the adsorption of the inhibitor on the carbon steel surface. The difference between *E*_corr_ values measured in the absence and presence of B1, B2, and B3 compounds is less than 85 mV, as shown in Table 1. As a result, these compounds block both the cathodic (hydrogen evolution) and anodic (metal dissolution) processes, acting as a mixed-type inhibitor.^58^ The slopes of the anodic (*β*_a_) and cathodic (*β*_c_) Tafel lines are slightly altered with the variety of inhibitor concentrations. This implies that the molecules B1, B2, and B3 influence the mechanism of carbon steel dissolution, and the *i*_corr_ values calculated from both the cathodic and anodic curves decreased by forming a protective layer against acid attack.

#### 3.2.2. EFM measurements

**Fig. 6:**
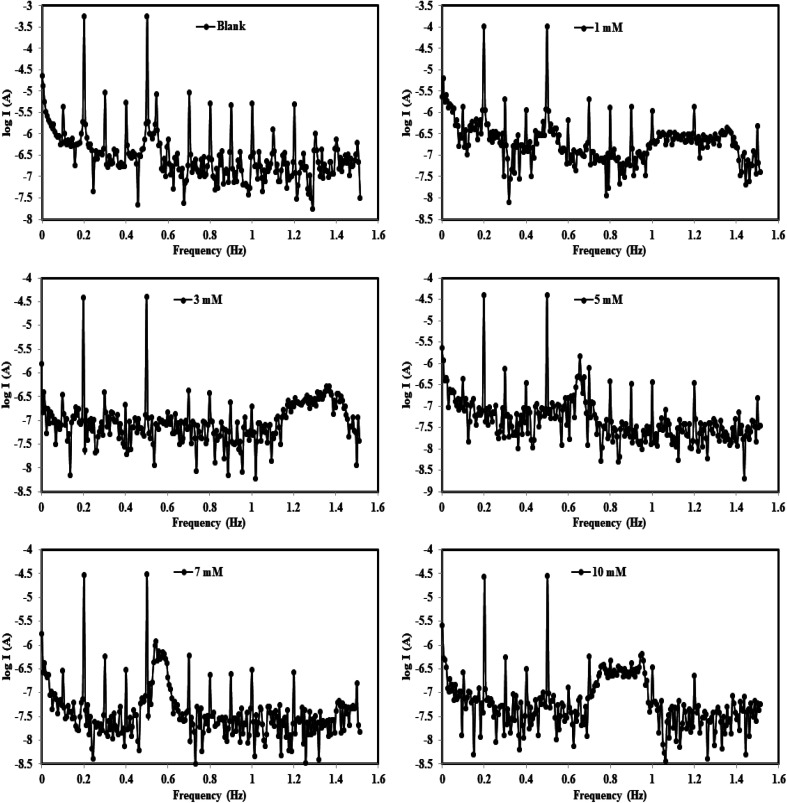
Intermodulation spectra for carbon steel in 1.0 M HCl in absence and presence of different concentrations from B3 compound at 30 °C.

EFM is well known as a quick and effective non-destructive corrosion rate assessment method.^59^ Without prior knowledge of Tafel constants, the EFM approach instantly yields corrosion current values.^60^ The intensity of the two higher peaks in the intermodulation spectra show the link between current density (i) and frequency in respect to the selected two applied excitation frequencies of 2 and 5 Hz.^61^ The EFM-intermodulation spectra of (X56 c-steel/1.00 M HCl/B3) at 30 °C are gathered in Fig. 6. In addition, the extracted electrochemical kinetic parameters are recorded in Table 2. The consistency between perturbation and response signals is indicated by the CF2 and CF3 values which are close to their ideal values of 2 and 3.^62^Fig. 6 depicts the sequentially suppressed spectra associated with the decrease in *i*_corr_ while B3 concentration increases. The corrosion rate dramatically decreased from 1211.0 mpy to 59.49 mpy, 38.07 mpy and 12.47 mpy by the addition of 10 mM of B1, B2 and B3, respectively. Also, a slight change in Tafel slopes values (*β*_c_ and *β*_a_) is noticed by the addition of B1, B2 and B3 compounds in agreement with data from PDP measurements. With the addition of 10 mM of B3, *i*_corr_ values dropped precipitously from 1077 μA cm^−2^ (blank) to 43.99 μA cm^−2^, producing protection efficiency of 95.92%. A similar behavior is observed for B2 and B1 inhibited c-steel specimens: *i*_corr_ decreased to 57.77 μA cm^−2^ and 85.02 μA cm^−2^ with a maximum efficiency of 94.64% and 92.11% respectively. The significant decrease of *i*_corr_ values articulates the creation of an organic adsorption layer at the X56 c-steel/1.00 M HCl interface by the order B3 > B2 > B1.

**Table 2:** Electrochemical kinetic parameters obtained by EFM technique for carbon steel in the absence and presence of various concentrations of (B1, B2 and B3) inhibitors in 1.0 M HCl at 30 °C^a^

Inhibitor name	Conc. (mM)	*i*_corr_ (μA cm^−2^)	*β*_a_ (mV dec^−1^)	*β*_c_ (mV dec^−1^)	CF-2	CF-3	*k* (mpy)	*θ*	*η*_EFM_ (%)
Blank	—	1077	117.8	133.7	1.712	3.021	499.2	—	—
B1	1	454.1	67.62	104.5	1.902	2.972	210.5	0.5784	57.84
	3	251.7	84.56	101.6	2.09	3.205	116.7	0.7663	76.63
	5	144.7	73.26	139.7	2.12	2.977	67.07	0.8656	86.56
	7	101.5	79.95	128.1	1.902	2.555	47.03	0.9058	90.58
	10	85.02	79.61	127.5	1.947	2.85	39.4	0.9211	92.11
B2	1	238.5	89.86	100.9	1.952	2.127	110.6	0.7786	77.86
	3	183.5	96.56	110.1	1.989	2.783	85.03	0.8296	82.96
	5	119.4	99.21	114.5	2.049	2.852	55.32	0.8891	88.91
	7	80.98	98.81	138.7	1.984	3.045	37.53	0.9248	92.48
	10	57.77	122.1	174.5	2.029	3.368	26.78	0.9464	94.64
B3	1	162.7	95.74	108.2	1.817	2.366	75.39	0.8489	84.89
	3	80.12	128.1	139.5	1.982	3.64	37.13	0.9256	92.56
	5	73.51	111.8	129.0	2.158	2.429	34.07	0.9317	93.17
	7	58.84	116.5	135.0	1.952	1.445	27.27	0.9454	94.54
	10	43.99	97.34	111.0	1.729	3.677	20.39	0.9592	95.92

a *E*_corr_, is the corrosion potential; *i*_corr_, is the corrosion current density: *β*_a_ and *β*_c_ are Tafel constants for both anode and cathode; *k*, is the corrosion rate; *θ*, is the surface coverage; *η*_EFM_, is the inhibition efficiency.

#### 3.2.3. EN measurements

**Fig. 7:**
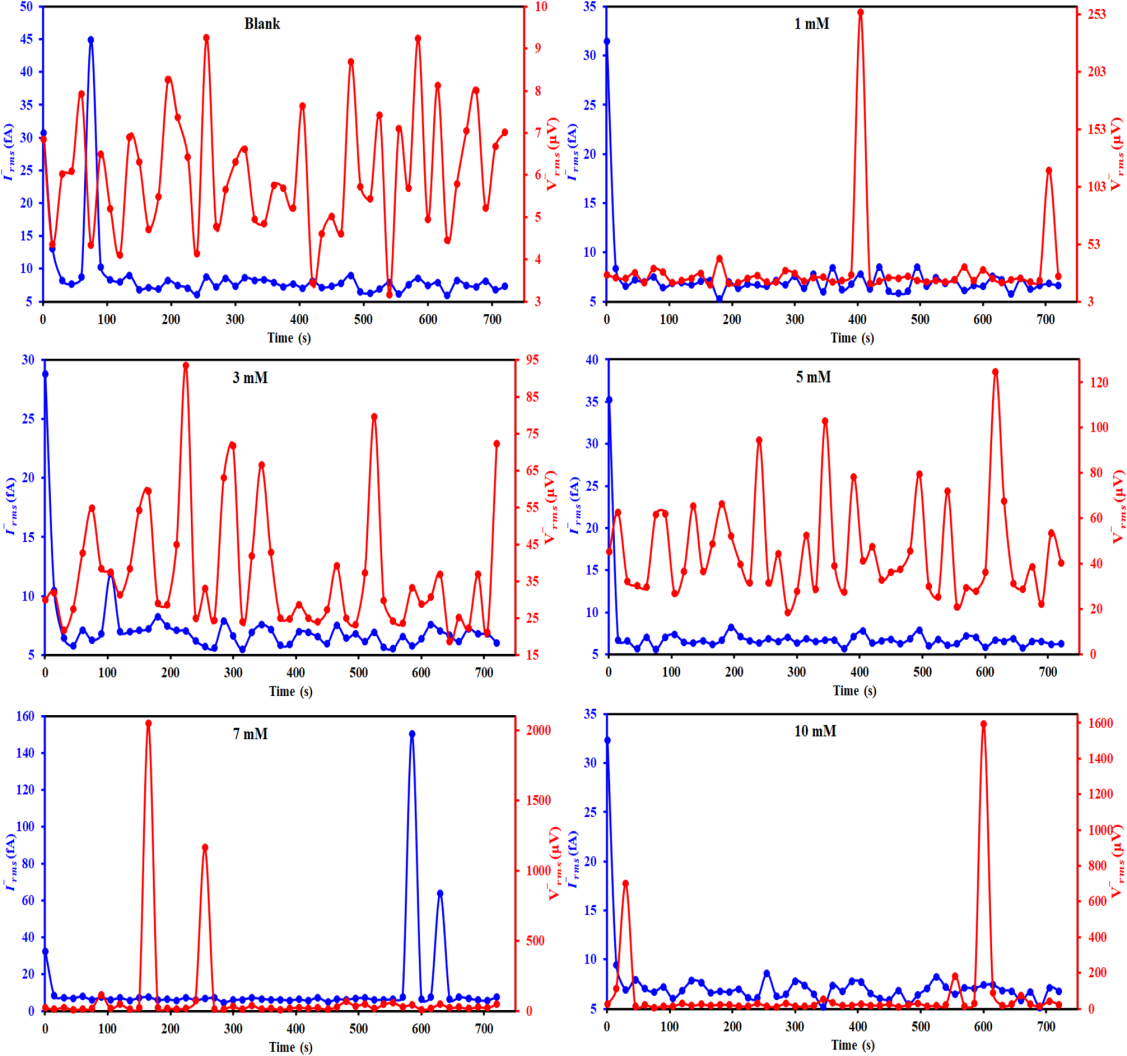
Electrochemical noise spectra for carbon steel in 1.0 M HCl in absence and presence of different concentrations from B3 compound at 30 °C.

The relationship between time and electrochemical noise (current and voltage), for the blank and B3 molecule at five different concentrations (1, 3, 5, 7 and 10 mM), is shown in Fig. 7. The extracted parameters for EN data are collected in Table 3. It is apparent that the 1 M HCl blank solution has more positive voltage and current areas with fluctuating potential and noise than the solution with B3 added. For the blank solution, the potential and current noise was stabilized at 6.02 μV and 8.981 fA, respectively. Due to their poor noise resistance, the H^+^ and Cl^−^ ions enhance the values of potential and current noise on the X56 c-steel surface. They have the ability to corrode X56 c-steel surfaces, and during EN tests, H_2(g)_ is seen on the X56 c-steel surfaces. On the other hand, the B1, B2 and B3 compounds cause the electrochemical noise fluctuation to move to more negative areas. This indicates that the B1, B2 and B3 compounds significantly reduces the corrosion current and potential.^63^ The change in voltage and current depends on B1, B2 and B3 concentrations. For example, in presence of B3 compound, the potential stabilizes at 30.15 μV at 1 mM, 37.11 μV at 3 mM, 45.80 μV at 5 mM, 94.59 μV at 7 mM and 74.62 μV at 10 mM. The inhibitors efficiencies were estimated as a function of resistance 
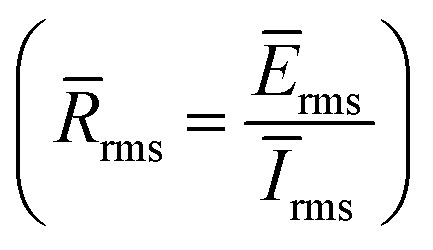
. The value of *R̄*_rms_ in the blank solution was 0.671 GΩ and enhanced by the successive addition of B1, B2 and B3 compounds. For example, in presence of B3 compound, *R̄*_rms_ values were 4.093 GΩ at 1 mM, 5.100 GΩ at 3 mM, 6.354 GΩ at 5 mM, 8.408 GΩ at 7 mM and 10.065 GΩ at 10 mM. At 10 mM and the efficiency approached 91.25% for B1, 92.55% for B2 and 93.34% for B3. The highest value of electrical charges (*q*) transferred between anode and cathode was related to the blank solution (6.294 pC) as a result of high corrosion rate.^64^ While in presence of B1, B2 or B3, its value reduced by blocking the active sites for electrical charges transfer leads to more corrosion resistance.

**Table 3:** Electrochemical parameters for steel dissolution in 1.0 M HCl solution containing different concentrations of the (B1, B2 and B3) inhibitors obtained from electrochemical noise (EN) measurements at 30 °C

Inhibitor name	Conc. (mM)	*Ē*_rms_ (μV)	*Ī*_rms_ (fA)	*R̄*_rms_ (GΩ)	*q* (pC)	*θ*	*η*_N_ (%)
Blank	—	6.02	8.981	0.671	6.294	—	—
B1	1	28.20	8.726	3.232	6.093	0.7925	79.25
	3	27.34	7.356	3.717	5.101	0.8196	81.96
	5	32.80	7.182	4.567	5.003	0.8532	85.32
	7	46.16	7.565	6.102	5.228	0.8901	89.01
	10	54.11	7.063	7.661	4.866	0.9125	91.25
B2	1	25.85	7.051	3.666	4.879	0.8171	81.71
	3	37.40	8.240	4.539	5.617	0.8522	85.22
	5	38.42	7.389	5.200	5.128	0.8710	87.10
	7	50.68	7.150	7.088	4.958	0.9054	90.54
	10	69.23	7.692	9.000	5.348	0.9255	92.55
B3	1	30.15	7.367	4.093	5.110	0.8361	83.61
	3	37.11	7.277	5.100	5.068	0.8685	86.85
	5	45.80	7.208	6.354	4.972	0.8945	89.45
	7	94.59	11.250	8.408	7.951	0.9202	92.02
	10	74.62	7.414	10.065	5.135	0.9334	93.34

#### 3.2.4. EIS measurements

**Fig. 8:**
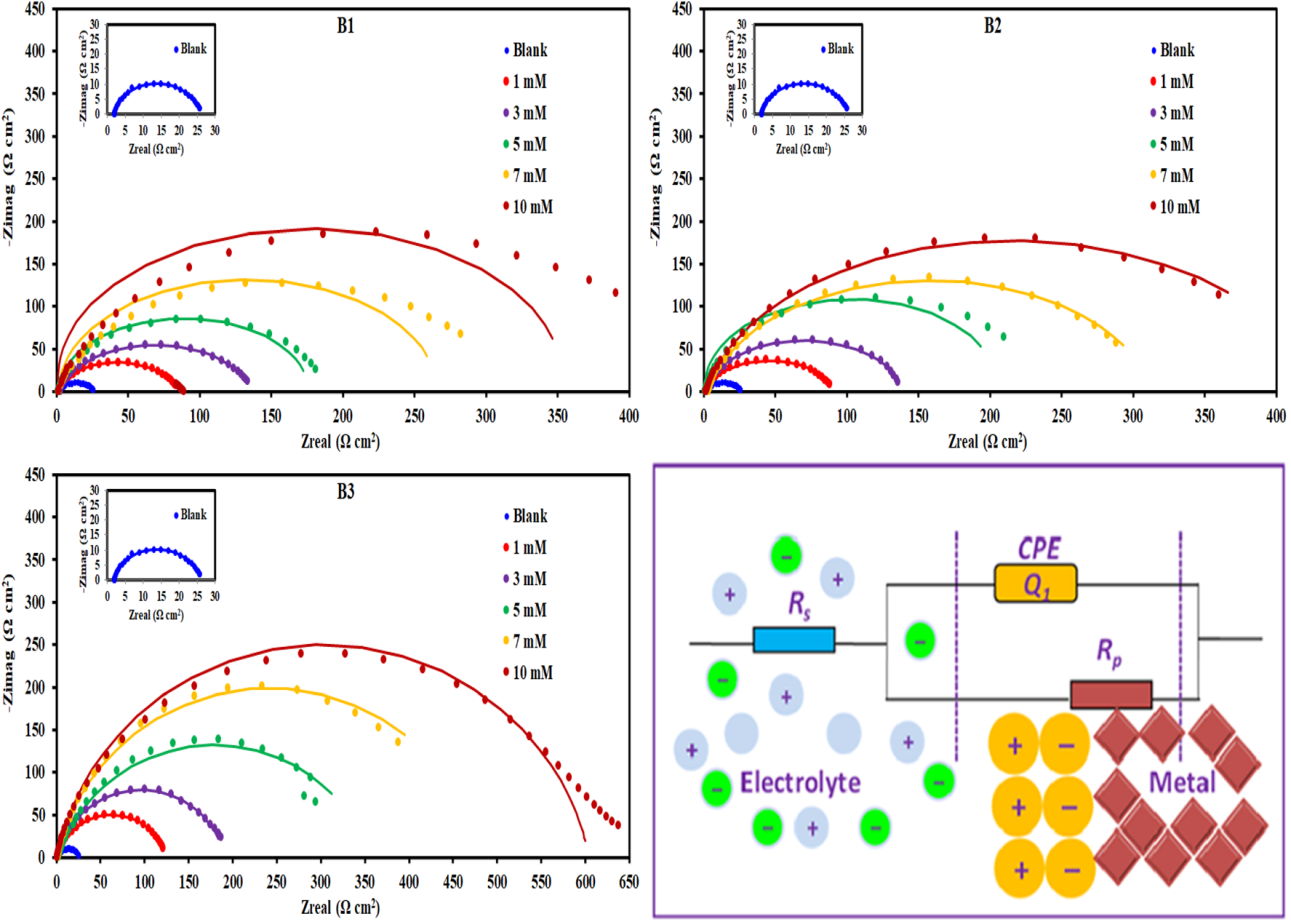
Nyquist plots and equivalent circuit for c-steel in 1.0 M HCl solution without and with different concentrations of B1, B2 and B3 compounds at 30 °C.

**Fig. 9:**
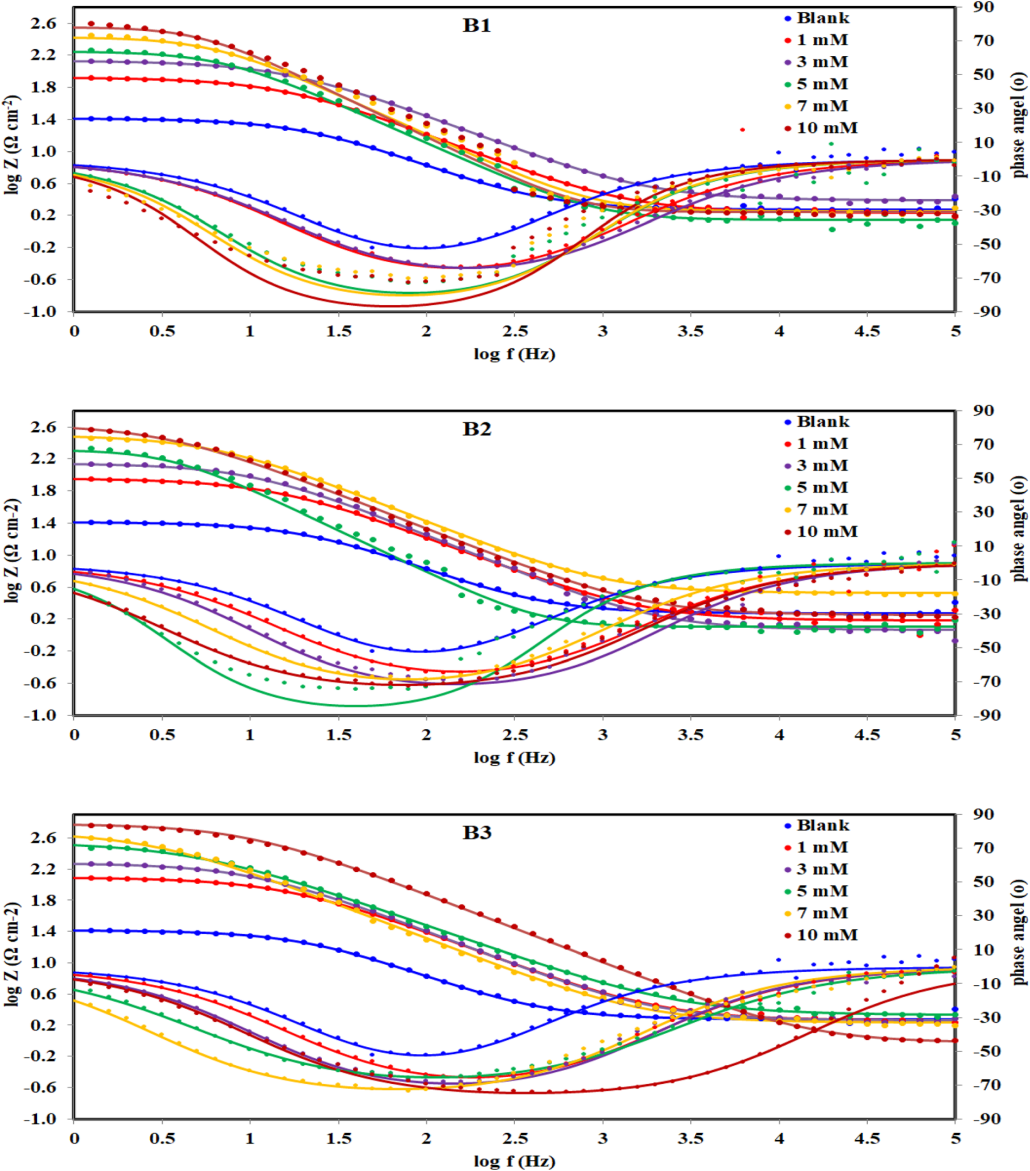
Bode and phase angle plots for c-steel in 1.0 M HCl solution without and with different concentrations of B1, B2 and B3 compounds at 30 °C.

Fig. 8 displayed Nyquist plots and the equivalent circuit for X56 c-steel surface in 1.0 M HCl solution containing B1, B2 and B3 molecules at 30 °C. The related Bode and phase angle plots are collected in Fig. 9. In Nyquist diagrams, there is just one depressed semi-circle (related to one time constant) which symbolizes charge-transfer controlled corrosion pathway.^65^ Due to the irregularity and roughness of the adsorbed constituent and X56 c-steel surface, Nyquist plots do not depict perfect semicircles.^66^ The presence of the B1, B3 and B3 compounds leads to increase of the semi-circle diameter as a function of concentration with priority of B3 over B2 and B1. The impedance data was fitted to a suitable equivalent circuit (*R*_s_ (CPE *R*_ct_)) and the extracted data were collected in Table 4. *R*_ct_ represents all resistance on X56 c-steel surface either charge transfer or organic film formation (sometimes expressed as *R*_p_) while *R*_s_ is the resistance of the aqueous solution. CPE (constant phase element) is usually used to express the non-perfect capacitance behavior.^67^ The reality of fitting to the selected equivalent circuit is indicated from the low values of *χ*^2^. The value of *R*_ct_ in the blank solution was the lowest (24.12 Ω cm^2^) and enhanced by the gradual addition of B1, B2 and B3 compounds due to their adsorption. For example, in presence of B3 compound, *R*_ct_ values were 121.1 Ω cm^2^ at 1 mM, 188.4 Ω cm^2^ at 3 mM, 3535.6 Ω cm^2^ at 5 mM, 479.2 Ω cm^2^ at 7 mM and 603.8 Ω cm^2^ at 10 mM. Higher *R*_ct_ values reflect excellent protection efficiency. At 10 mM, the efficiency approached 93.11% for B1, 94.36% for B2 and 96.01% for B3. The double layer capacitance is estimated using *R*_ct_ and the CPE constants (*Y*_0_, *n*) by the equation:^68^6
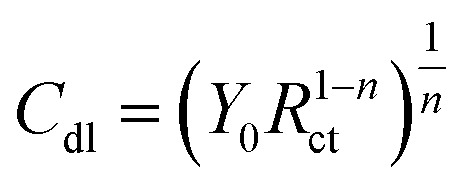


**Table 4:** EIS parameters for corrosion of steel in 1.0 M HCl in the absence and presence of different concentrations of (B1, B2 and B3) inhibitors at 30 °C^a^

Inhibitor	Conc. (mM)	*R*_s_ (*R*_u_) (Ω cm^2^)	*R*_ct_ (*R*_p_) (Ω cm^2^)	*Y*_0_ (μΩ^−1^ s^*n*^ cm^−2^)	*n*	*C*_dl_ (μF cm^−2^)	Chi squared (*χ*^2^)	*τ* (mS)	*S*	*α*°	*θ*	*η*_*z*_ (%)
Blank	—	1.880	24.12	492.40	0.8987	298.745	3.52 × 10^−3^	7.206	−0.568	−52.31	—	—
B1	1	1.698	81.94	204.70	0.8847	120.154	8.69 × 10^−3^	9.845	−0.725	−63.59	0.7056	70.56
	3	2.444	133.6	127.50	0.8723	70.240	1.15 × 10^−3^	9.384	−0.727	−64.55	0.8195	81.95
	5	1.379	174.7	134.40	0.9908	129.799	2.58 × 10^−2^	22.676	−0.951	−73.32	0.8619	86.19
	7	1.785	263.9	98.36	0.9994	98.145	3.42 × 10^−2^	25.900	−0.924	−70.85	0.9086	90.86
	10	1.758	350.3	73.01	1.056	88.678	5.99 × 10^−2^	31.064	−1.071	−73.13	0.9311	93.11
B2	1	1.500	88.89	221.00	0.8705	123.165	1.22 × 10^−2^	10.948	−0.743	−65.27	0.7287	72.87
	3	1.160	138.1	161.70	0.9073	109.652	1.02 × 10^−2^	15.143	−0.838	−69.36	0.8253	82.53
	5	1.276	203.4	197.00	1.043	224.941	3.51 × 10^−2^	45.753	−0.939	−74.93	0.8814	88.14
	7	3.338	310.0	123.90	0.8915	83.328	1.14 × 10^−3^	25.832	−0.796	−68.93	0.9222	92.22
	10	1.779	427.4	160.60	0.8814	111.995	4.30 × 10^−3^	47.867	−0.841	−73.07	0.9436	94.36
B3	1	1.828	122.1	142.00	0.8745	79.354	9.55 × 10^−4^	9.689	−0.741	−66.02	0.8025	80.25
	3	1.816	188.4	127.30	0.8899	80.239	2.81 × 10^−3^	15.117	−0.787	−68.35	0.8720	87.20
	5	2.135	353.6	170.40	0.8183	91.321	2.42 × 10^−3^	32.291	−0.759	−66.06	0.9318	93.18
	7	1.724	479.2	165.30	0.8819	117.707	6.75 × 10^−3^	56.405	−0.846	−74.10	0.9497	94.97
	10	0.947	603.8	44.74	0.8811	27.4819	4.59 × 10^−3^	16.594	−0.804	−74.32	0.9601	96.01

a *R*_s_ = solution resistance, *R*_ct_ = charge transfer resistant, *Y*_0_, *n* = constant phase elements, *C*_dl_ = double layer capacitance, *θ* = surface coverage, *η*_*z*_ = inhibition efficiency.

The values of *Y*_0_ and *C*_dl_ in the blank solution were high (492.40 μΩ^−1^ s^*n*^ cm^−2^ and 298.745 μF cm^−2^) and decreased by the presence of B1, B2 and B3 compounds. As *C*_dl_ is inversely related to the double layer thickness, so the depression in *C*_dl_ values is associated with increasing the double layer thickness by replacing the adsorbed water, H^+^ and Cl^−^ chemical species with the organic B1, B2 and B3 molecules.^69^ The other proof of the adsorbed film of B1, B2 and B3 molecules on the X56 c-steel surface is the close values of *n* to unity in the presence of B1, B2 and B3 molecules.

The Bode plots (Fig. 9) consist of one phase that belongs to one time constant. Generally, higher corrosion resistance is associated with a bigger capacitive loop at |*Z*|_0.01Hz_. Moreover, a stronger corrosion barrier layer was shown by the greater and broader phase angle in the middle frequency band.^70^ The value of |*Z*|_0.01Hz_ in the blank solution was the lowest and enhanced by the gradual addition of B1, B2 and B3 compounds to achieve almost 2 orders of magnitude greater than that of the blank, demonstrating good X56 c-steel protection. The phase angles are almost zero at high frequency confirming that the electrolyte exhibiting a low resistance as indicated by the equivalent circuit with the solution resistance (*R*_s_) and the beginning of the adsorption process. On the other hand, the increase in the phase angles at the low-frequency regions confirms the better corrosion inhibition with B1, B2 and B3 indicating that the inhibitors molecules get adsorbed on the surface of steel and forming a protective film.^71^ Furthermore, when B1, B2 and B3 molecules present, the phase angle expands (towards −90), demonstrating their inhibitory impact on X56 c-steel.^72^ The phase angle for the blank is −52.31 and at 10 mM; it was shifted to −73.13 (B1), −73.07 (B2) and −74.32 (B3). The Bodes lines slopes at the middle frequency range shift to −1 in presence of B1, B2 or B3 compounds as indication of enhanced capacitance behavior. The relaxation time (*τ*) was calculated as the product of *R*_ct_ × *C*_dl_,^73^ and listed in Table 4. The addition of the investigated molecules increased the values of *τ* from 7.206 mS to 31.064 mS (B1), 47.867 mS (B2) and 56.405 mS (B3), which may be explained on the basis that building up dense layers of B1, B2 and B3 molecules on X56 c-steel need more time for adsorption, delaying the adsorption process.^74^

### 3.3. Adsorption considerations

**Fig. 10:**
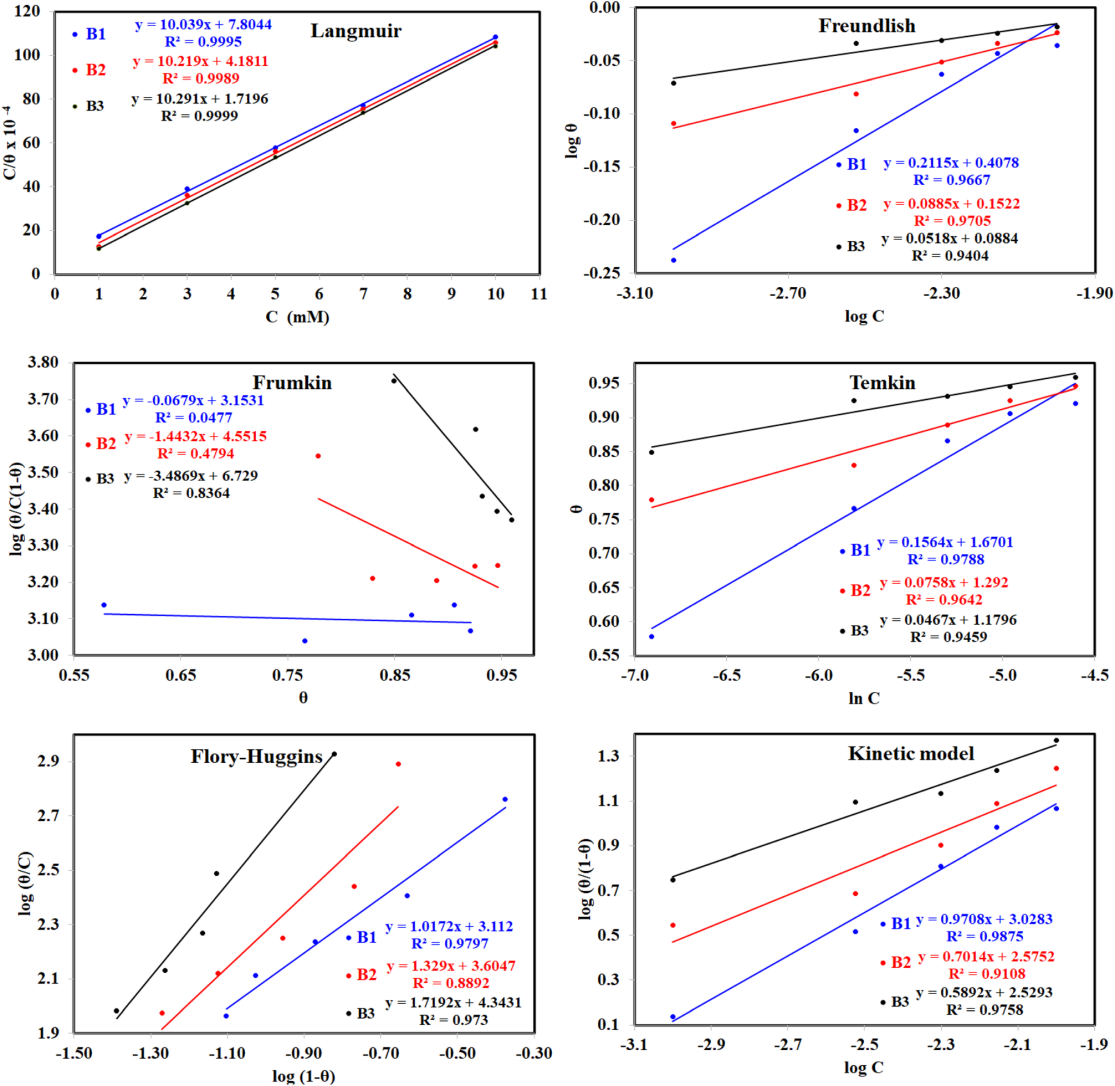
The different adsorption models for B1, B2 and B3 compounds on the steel surface in 1.0 M HCl using data obtained from EFM measurements at 30 °C.

The interaction of B1, B2 and B3 molecules with X56 c-steel surface could be physical or chemical forming single layer or multiple layers. The data from EFM were subjected to several adsorption isotherms to select the most suitable one. The graphical representations of six adsorption models were collected in Fig. 10 and the extracted fitting parameters were collected in Table 5. Based on *R*^2^ values (*R*^2^ ≈ 1),^75^ we can rank the fitted isotherms as: Langmuir (*R*^2^ = 0.99989) > kinetic-thermodynamic (*R*^2^ = 0.97577) > Flory–Huggins (*R*^2^ = 0.97304) > Temkin (*R*^2^ = 0.94591) > Freundlich (*R*^2^ = 0.94 045) > Frumkin (*R*^2^ = 0.83642). Based on Langmuir model (most suitable isotherm), the binding constant (*K*_ads_) can be calculated by the equation:^76^7
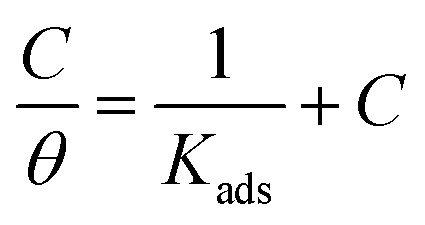


**Table 5:** Adsorption isotherms models of the inhibitors with values of *R*^2^, slopes, intercepts, *K*_ads_, and Δ*G*_ads_ obtained by using data from electrochemical measurements^a^

Adsorption isotherm model	Linear form equation	Technique	Inhibitor	Slope	Intercept	*R* ^2^	*K*_ads_ (M^−1^)	Δ*G*_ads_ (kJ mol^−1^)
Freundlich	log *θ* = log *K* + 1/*n* log *C*	EFM	B1	0.21153	0.40784	0.96667	2.5577	−12.48
			B2	0.08845	0.15216	0.97045	1.4196	−11.00
			B3	0.05177	0.08844	0.94045	1.2258	−10.63
Langmuir	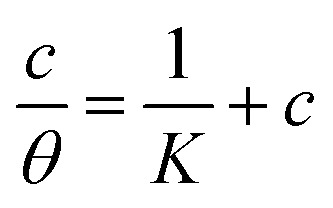	EFM	B1	1.00394	0.00078	0.99946	1281	−28.14
			B2	1.02189	0.00042	0.99890	2392	−29.72
			B3	1.02906	0.00017	0.99989	5815	−31.95
		EIS	B1	1.02978	0.00051	0.99915	1961	−29.22
			B2	1.01714	0.00048	0.99920	2064	−29.34
			B3	1.01202	0.00031	0.99969	3276	−30.51
		EN	B1	1.07037	0.00036	0.99876	2815	−30.13
			B2	1.06040	0.00029	0.99911	3447	−30.64
			B3	1.05281	0.00024	0.99958	4210	−31.14
		PDP	B1	0.99696	0.00066	0.99872	1521	−28.58
			B2	0.97728	0.00061	0.99864	1627	−28.75
			B3	0.98175	0.00035	0.99945	2870	−30.18
Frumkin	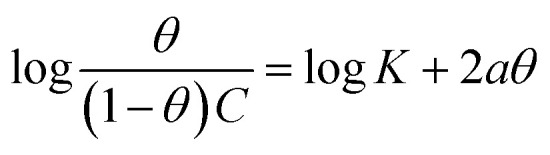	EFM	B1	−0.06794	3.15306	0.04771	1.4225 × 10^3^	−28.41
			B2	−1.44317	4.55152	0.47937	3.5605 × 10^4^	−36.52
			B3	−3.48687	6.72901	0.83642	5.3581 × 10^6^	−49.15
Temkin	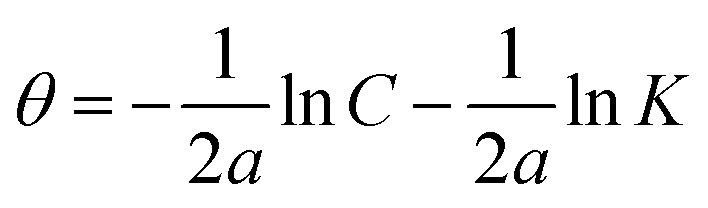	EFM	B1	6.25900	−10.57022	0.97880	0.1847	−5.86
			B2	12.71647	−16.62683	0.96423	0.2705	−6.82
			B3	20.27165	−24.21015	0.94591	0.3029	−7.11
Flory–Huggins	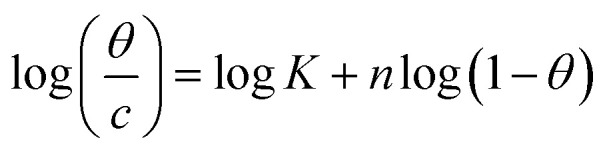	EFM	B1	1.01723	3.11201	0.97972	1.2942 × 10^3^	−28.17
			B2	1.32900	3.60469	0.88924	4.0243 × 10^3^	−31.03
			B3	1.71924	4.34311	0.97304	2.2035 × 10^4^	−35.31
Kinetic-thermodynamic	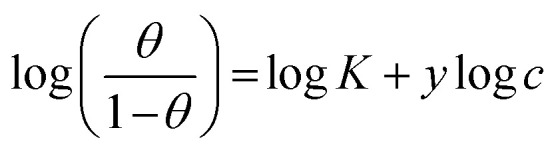	EFM	B1	0.97081	3.02828	0.98746	1067.2762	−27.68
			B2	0.70136	2.57516	0.91081	375.9775	−25.06
			B3	0.58917	2.52931	0.97577	338.3063	−24.79

a *R* ^2^ = regression correlation coefficient, *K* = binding constant, *θ* = surface coverage, *c* = concentration.

Large values of binding constant (5815 M^−1^ for B3 > 2392 M^−1^ for B2 > 1281 M^−1^ for B1) imply a strong adsorption. In addition, the characteristics of Langmuir model were verified as the adsorption is mono layer and the active sites are identically occupied by equivalent number of organic molecules.^77^ The nature of binding as physical or chemical could be confirmed from the values of free energy (Δ*G*_ads_) which is estimated by the equation:^78^

8 Δ*G*_ads_ = −*RT* ln 55.5*K*_ads_

(*R* = 8.314 J mol^−1^ K^−1^, *T* = 303.15 K, [H_2_O] = 55.5 mol L^−1^)Δ*G*_ads_ values ranged from −28.14 kJ mol^−1^ to −31.14 kJ mol^−1^ as indication of spontaneous collaborative mode of physical and chemical adsorption (−20 kJ mol^−1^ physisorption > collaborative or mixed mode > −40 kJ mol^−1^ chemisorption).^79^

### 3.4. FESEM analysis

**Fig. 11:**
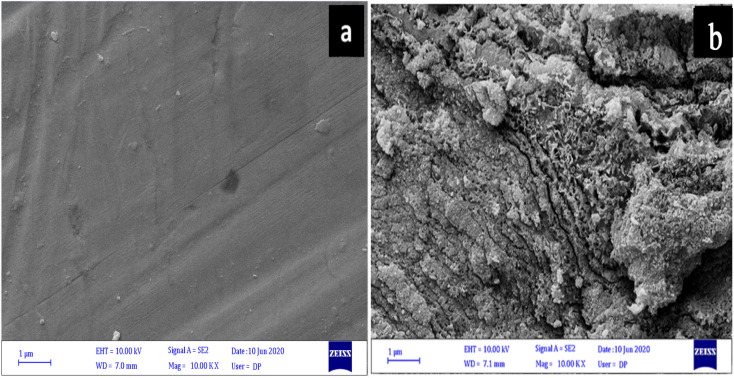
SEM images of the X56 carbon steel surface (a) polished and (b) after 24 hours immersion in 1.0 M HCl.

**Fig. 12:**
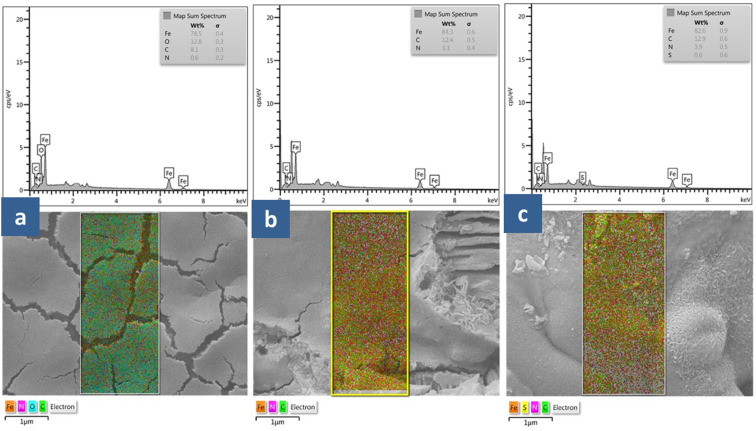
SEM images and EDX mapping for the distribution of the elements on the surface of X56 carbon steel coupon after 24 hours immersion in 1.0 M HCl in presence of 10 mM of B1 (a), B2 (b) and B3 (c).

The surface morphology of carbon steel samples immersed for 24 hours in 1.0 M HCl with and without B1, B2, and B3 inhibitors was analyzed using FESEM. As shown in Fig. 11a, the surface of the carbon steel was polished before it was immersed in a corrosive acidic solution. Inevitably, removing corrosion with a succession of silica carbide sheets will leave visible scratches on the surface of the carbon steel. These scratches cannot be prevented. Fig. 11b shows a blank FESEM micrograph of the metal-carbon surface immersed in an acidic solution of 1.0 M HCl. In absence of an inhibitor, metal corrosion would cause much damage to the surface of the specimen. It is clear to see many pits of different sizes and depths all over the carbon steel surface exposed to an acidic corrosive. This shows that the corrosion process has made much damage. The surface became rough because of dissolution in the corrosive media. Furthermore, a thick porous layer of corrosion products like oxide films covered all surface metal, and the surface was strongly damaged.^80^ The damage on the specimen surface was significantly reduced after the addition of B1, B2, and B3 corrosion inhibitors (10 mM) to the corrosive solution, as shown in Fig. 12a–c, because the pits and cracks were not as aggressive as the previous ones, as shown in Fig. 11b. The image of FESEM for carbon steel in 1.0 M HCl with 10 mM B3 shows that some parts had smoother surfaces and fewer cracks. However, in case of B1 inhibitor, the carbon steel's protective layer seems cracked while B2 inhibitor can be uniform and stick to the surface metal. It was confirmed that the B1, B2, and B3 inhibitors work well as inhibitors because they can form a protective layer by adsorption that can protect the metal surface from a corrosive environment. Thus, the presence of B1, B2, and B3 compounds can enhance the surface morphology of carbon steel. They are effective corrosion inhibitors by decreasing the interaction between the metal surface and the corrosive solution.

We performed an EDX analysis to support these results further. EDX analysis reveals the semi-quantitative distribution of elements on the surface of carbon steel. The findings are presented in Fig. 12 and Table 6. After 24 hours in 1.0 M HCl with 10 mM of inhibitors, the elemental distribution on the surface of the carbon steel was analyzed by EDX (Fig. 12). Surface elemental composition after 24 hours in 1 M HCl displayed another peak, indicative of sulphur (S) element, as shown in Fig. 12c. The EDX spectra of B1 revealed a small amount of N by weight, but the 12.8% O composition was easily discernible (Table 6). EDX mapping images show the relative abundance of O and N elements. The corrosion reaction was slowed because of the presence of N, O, and S atoms, which served as active centers for adsorption and film formation on the metal surface according to the structure of inhibitors.

**Table 6:** Quantitative analysis for X56 carbon steel surface after 24 h immersion in 1.0 M HCl in presence of the investigated compounds (B1, B2 and B3) obtained from EDX

Element	B1/HCl/steel		B2/HCl/steel		B3/HCl/steel	
	Mass (%)	Atom (%)	Mass (%)	Atom (%)	Mass (%)	Atom (%)
C K	8.1	23.1	12.4	37.2	12.9	37.7
N K	0.6	1.5	3.3	8.5	3.9	9.8
O K	12.8	27.4	—	—	—	—
S K	—	—	—	—	0.6	0.7
Fe K	78.5	48.1	84.3	54.3	82.6	51.9
Total	100	100	100	100	100	100

### 3.5. Molecular reactivity

#### 3.5.1. Global reactivity

The electrons lost by metals in a corrosion reaction can be compensated by adsorption of organic inhibitors on the metal surface so that the reaction can be controlled. The adsorption should guarantee an ease electron flow from the inhibitor to the metal surface. Molecules with low band gap, low ionization potential and low chemical hardness are the best candidates for such effective adsorption.^81^ Although the band gap can be calculated directly by subtracting the eigenvalue of HOMO from that of LUMO, common DFT methods don't predict both HOMO and LUMO eigenvalues correctly resulting in an inaccurate band gap. Following the procedure suggested by Zhang and Musgrave *et al.*^54^ an accurate band gap can be obtained using time dependent (TD) calculations.

A correction to HOMO can be calculated from the following relation:

9 −HOMO_corr_ = *A* + *B*(−HOMO_cal_)

A correction to LUMO can be calculated from the following relation:

10 LUMO = HOMO + Δ*E*_gap_

**Fig. 13:**
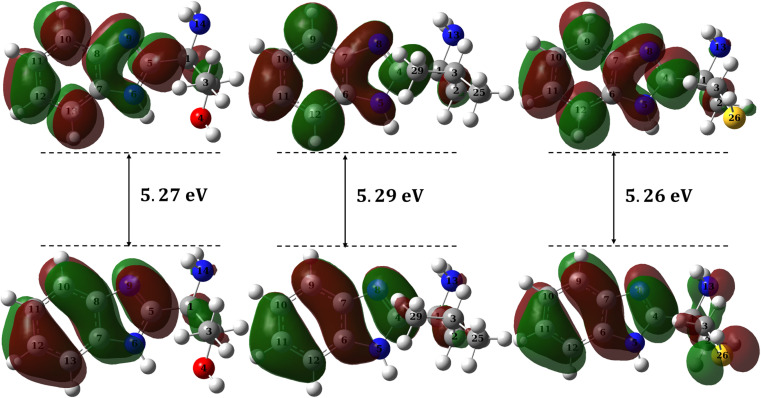
HOMO and LUMO densities for B1 (left), B2 (middle), B3 (right).

The calculated eigenvalues of both HOMO and LUMO are presented in Table 7 along with the corresponding global reactivity indices while their densities are plotted in Fig. 13. The estimated Δ*E*_gap_ calculated according to Zhang and Musgrave *et al.*^54^ procedure for B1, B2 and B3 molecules are 5.27, 5.29 and 5.26 eV, respectively (Table 7). The calculated narrow band gaps reveal a smooth polarization between HOMO and LUMO and therefore an effortless excitation is expected from HOMO to LUMO. No doubt, this soft mix of wavefunctions guarantees an ease flow of electrons from the inhibitor to the metal and therefore an effective electron compensation to the corrosion reaction. Moreover, B1, B2 and B3 molecules show low ionization potentials which are 8.45, 8.59 and 8.74 eV, respectively (Table 7) revealing an ease electron share when adsorbed to metallic surface. The corresponding orbital densities of HOMOs of B1, B2 and B3, respectively are mainly localized on the π-system of the phenyl ring in addition to the CN double bond of the imidazole ring (Fig. 13). However, no contribution, from hetero atoms (N, O and S atoms) to the HOMO of either B1 or B2, is noticed with a low contribution to HOMO of B3 molecule though. The lopes of the HOMO and LUMO for B1, B2 and B3 molecules are very close in size and shape reflecting the proximity of molecular structure of these molecules and hence accounting for the obtained close inhibition efficiencies to the corrosion of mild steel in HCl solution. Moreover, synthesized molecules with low chemical hardness (1.94 ± 1; Table 7), are good adsorbates forming stable films on the adsorbent which is the metal in this case. In addition, the electron flow from inhibitor to the metal can be estimated using the following function as suggested by Pearson:^82^11
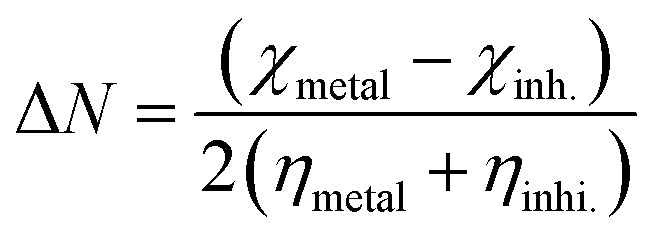
where *χ* and *η* are electronegativity and chemical hardness, respectively. The estimated Δ*N* for the B1, B2 and B3 molecules are 0.102, 0.075 and 0.041, respectively (Table 7) showing good flow of electrons from these molecules to the mild steel surface. Overall, calculated reactivity parameters reveal an ease and effortlessness electron share from the synthesized molecules to the metal surface which explains the highly obtained electrochemical inhibition efficiencies.

**Table 7:** Calculated electronic reactivity descriptors for B1, B2 and B3 molecules

Molecular Parameters^a^	B1		B2		B3	
	B3LYP	TD-B3LYP^b^	B3LYP	TD-B3LYP^b^	B3LYP	TD-B3LYP^b^
*E*_LUMO_	−0.21	−4.57	−0.34	−4.69	−0.47	−4.87
*E*_HOMO_	−5.86	−8.45	−5.98	−8.59	−6.1	−8.74
Δ*E* (*E*_LUMO_ − *E*_HOMO_)	5.65	5.27(3.88^c^)	5.64	5.29(3.9^c^)	5.63	5.26(3.87^c^)
Ionization potential (IP)	5.86	8.45	5.98	8.59	6.1	8.74
Electron affinity (EA)	0.21	4.57	0.34	4.69	0.47	4.87
Electronegativity (*χ*)	3.035	6.51	3.16	6.64	3.285	6.805
Chemical potential (*μ*)	−3.035	−6.51	−3.16	−6.64	−3.285	−6.805
Chemical hardness (*η*)	2.825	1.94	2.82	1.95	2.815	1.935
Chemical softness (*σ*)	0.354	0.515	0.355	0.513	0.355	0.517
Global electrophilicity index (*ω*)	1.63	10.923	1.77	11.305	1.917	11.966
Δ*N*	0.605	0.102	0.587	0.075	0.569	0.041

a All parameters in eV except Δ*N* is dimensionless quantity.

b All values are corrected according to the procedure in ref. 48.

c The lowest excitation energy before correction.

#### 3.5.2. Natural bond orbital analysis

**Fig. 14:**
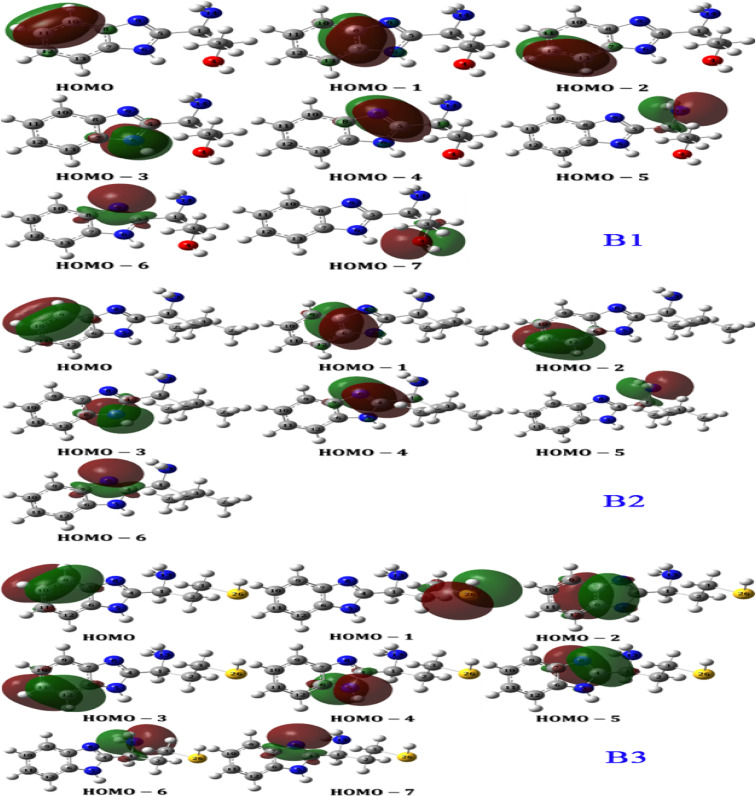
NBOs densities for B1, B2 and B3 molecules calculated at B3LYP/6-31g(d,p) level.

Table 8 lists NBOs of the orbitals of B1, B2 and B3 molecules from HOMO to HOMO-7 which are the expected sites of interaction with metal surface. The list includes the type of orbital, its population, energy, NBO and hybridization limited to s and *p* characters. Furthermore, the orbital densities are sketched and included in Fig. 14 by the same order. NBOs order suggest that the orbital with higher energy (*i.e.* HOMO) should has the highest donating probability to the metal. Therefore, it resolves the HOMO of the FMOs into individual bond by bond orbital ordered according to their donating priority. Accordingly, the electron flow from the inhibitor B1 to the metal is as follows:BD (2) C_10_–C_11_ > BD (2) C_7_–C_8_ > BD (2) C_12_–C_13_ > LP (1) (N_6_) > BD (2) C_5_–N_9_ > LP (1) (N_14_) > LP (1) (N_9_) > LP (2) (O_4_)

**Table 8:** NBOs at inhibitor-metal interactions ordered according to their energies (highest to lowest)

	Type^a^	Occupancy	Energy	NBO	s % (Atom 1)	p % (Atom 1)	s % (Atom 2)	p % (Atom 2)
B1 molecule	BD(2)C_10_–C_11_	1.71812	−0.23088	0.6991 p + 0.7150 p	0.00	99.95	0.00	99.96
	BD(2)C_7_–C_8_	1.59062	−0.23147	0.7072 p + 0.7070 p	0.00	99.97	0.00	99.97
	BD(2)C_12_–C_13_	1.72931	−0.23650	0.6995 p + 0.7146 p	0.00	99.96	0.00	99.96
	LP(1)(N_6_)	1.61007	−0.25326	p^0.99^	0.70	99.28	—	—
	BD(2)C_5_–N_9_	1.87171	−0.29289	0.6305 p + 0.7762 p	0.01	99.89	0.00	99.70
	LP(1)(N_14_)	1.95306	−0.30523	sp^3.60^	21.72	78.21	—	—
	LP(1)(N_9_)	1.92267	−0.34709	sp^2.10^	32.25	67.60	—	—
	LP(2)(O_4_)	1.95789	−0.35146	sp^4.32^	6.52	93.41	—	—
B2 molecule	BD(2)C_9_–C_10_	1.71426	−0.23527	0.6993 p + 0.7148 p	0.00	99.95	0.00	99.96
	BD(2)C_6_–C_7_	1.59402	−0.23798	0.7080 p + 0.7062 p	0.00	99.97	0.00	99.97
	BD(2)C_11_–C_12_	1.72661	−0.24152	0.6985 p + 0.7156 p	0.00	99.96	0.00	99.96
	LP(1)(N_5_)	1.62867	−0.26322	p^1.00^	0.00	99.99	—	—
	BD(2)C_4_–N_8_	1.87140	−0.29783	0.6277 p + 0.7785 p	0.00	99.89	0.00	99.70
	LP(1)(N_13_)	1.95262	−0.30239	sp^3.52^	22.12	77.81	—	—
	LP(1)(N_8_)	1.92017	−0.35075	sp^2.10^	32.17	67.68	—	—
B3 molecule	BD(2)C_9_–C_10_	1.71422	−0.23927	0.6997 p + 0.7144 p	0.00	99.95	0.00	99.96
	LP(2)(S_26_)	1.97732	−0.23952	p^1.00^	0.00	99.96	—	—
	BD(2)C_6_–C_7_	1.59567	−0.24272	0.7079 p + 0.7063 p	0.00	99.97	0.00	99.97
	BD(2)C_11_–C_12_	1.72569	−0.24527	0.6984 p + 0.7157 p	0.00	99.96	0.00	99.96
	LP(1)(N_5_)	1.62697	−0.26868	p^1.00^	0.00	99.99	—	—
	BD(2)C_4_–N_8_	1.87402	−0.30448	0.6303 p + 0.7764 p	0.01	99.89	0.00	99.70
	LP(1)(N_13_)	1.95233	−0.30865	sp^3.54^	21.99	77.94	—	—
	LP(1)(N_8_)	1.91945	−0.35700	sp^2.11^	32.07	67.78	—	—

a LP(1): refers to first lone pair, LP(2): second lone pair, *etc.* BD(1): bonding orbital of a single bond, BD(2): for double bond.

Fig. 14 shows the origin of these bonding orbitals; for example, bonding orbital BD (2) C_10_–C_11_ belongs to the HOMO of B1 molecule. In other words, the HOMO of B1 molecule is localized on the double bond C_10_–C_11_ of the benzimidazole ring. In this order, the second lone pair of oxygen atom of the hydroxyl group comes late at HOMO−7 which indicates weaker ability to reallocate its density into empty orbitals of the metal. Similarly, the electron flow from inhibitor B2 has the following order (Table 8/Fig. 14):BD (2) C_9_–C_10_ > BD (2) C_6_–C_7_ > BD (2) C_11_–C_12_ > LP (1) (N_5_) > BD (2) C_4_–N_8_ > LP (1) (N_13_) > LP (1) (N_8_)

It is noticeable that B2 has the same donating sites with the same order as molecule B1 except that B2 doesn't have Oxygen atom. NBOs of B3 molecule reveal similar electron flow character as well (Table 8/Fig. 14):BD (2) C_9_–C_10_ > LP (2) (S_26_) > BD (2) C_6_–C_7_ > BD (2) C_11_–C_12_ > LP (1) (N_5_) > BD (2) C_4_–N_8_ > LP (1) (N_13_) > LP (1) (N_8_)

#### 3.5.3. Local reactivity

**Fig. 15:**
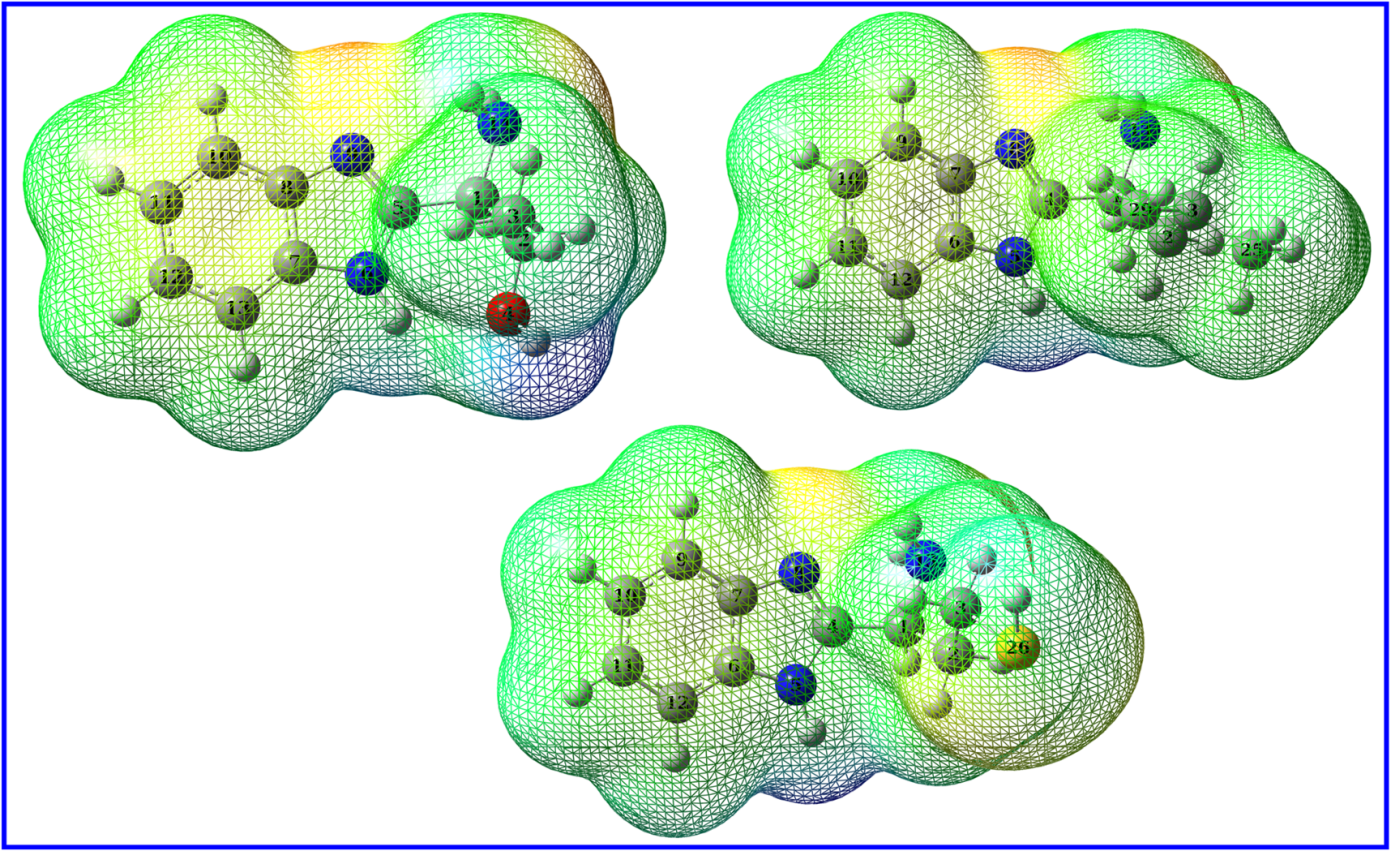
MEP densities for B1 (left), B2 (right), B3 (bottom).

While global reactivity descriptors relate the reactivity to the orbitals belongings, local reactivity concerns the sites (atoms, functional groups, and electrostatic surfaces) where the molecule can donate or accept electrons. A good inhibitor should have atoms or sites that are rich in electrons. A one way to explore these sites is the investigation of the molecular electrostatic potential (MEP) map (Fig. 15). In MEP, the sites with low electrostatic potential are rich in electrons while sites with high electrostatic potential are poor in electrons.^83^ The first one is characterized by red color and the latter by blue color while sites with green color points to intermediate potential. Fig. 15 show that the color content follows the order: green > red > blue. While widespread green sites are common in organic molecules, MEP maps of B1, B2 and B3 molecules are depicted with electron rich sites much more than electron poor ones though. The red regions are localized on the benzimidazole part of the molecule which agrees with that HOMO is localized on both phenyl and imidazole rings and agrees also with that NBOs suggests that benzimidazole part has the priority to contact metal surface. Although MEP map labels the donating sites within the molecule, the magnitude of this donation can't be assessed from MEP map. Instead, Fukui functions as a powerful tool to estimate the nucleophilic and electrophilic tendency of a particular atom. To do so, the molecule is positively and negatively charged so that the second derivative of energy is probed with respect to number of electrons and the external potential while molecules approaching each other as follow:

12 *f*(*r*) = ∂^2^*E*/∂*N*∂*ν*

A good approximation to solve this expression is the use of “condensed” Fukui functions on an atom-by-atom basis:^84^

13 *f*_*k*_^−^ = [*q*_*k*_(*N*) − *q*_*k*_(*N* − 1)]

14 *f*_*k*_^+^ = [*q*_*k*_(*N* + 1) − *q*_*k*_(*N*)]

where *f*_*k*_^−^ and *f*_*k*_^+^ are electrophilic and nucleophilic Fukui indices, respectively and *q*_*k*_ is the atomic charge. Fukui indices for the B1, B2 and B3 molecules are listed in Table 9 according to the descending order of electrophilic Fukui function. For molecule B1, the highest ninth electrophilic atoms are those of benzimidazole six and five-membered rings with Fukui indices between 0.101 and 0.023 then atoms N_14_(0.023), C_1_(0.019), C_2_(0.013), C_3_(0.009) and O_4_(0.005). Similar results were found for both B2 and B3 molecules and listed in Table 9 with the same manner. While these results match with those of NBOs and MEP map, Fukui indices are good estimation for the magnitude of interactions at specific atoms.

**Table 9:** Calculated condensed Fukui functions for B1, B2 and B3 molecules^a^

B1			B2			B3		
Atoms	*f* ^+^	*f* ^−^	Atoms	*f* ^+^	*f* ^−^	Atoms	*f* ^+^	*f* ^−^
C_10_	0.064	0.101	C_9_	0.066	0.101	C_9_	0.055	0.092
C_13_	0.053	0.098	C_12_	0.055	0.098	C_12_	0.048	0.089
C_5_	0.075	0.092	C_4_	0.074	0.095	C_4_	0.05	0.084
C_12_	0.114	0.083	C_11_	0.112	0.085	C_11_	0.087	0.079
N_9_	0.073	0.064	N_8_	0.075	0.065	S_26_	0.202	0.074
C_11_	0.058	0.056	C_10_	0.055	0.055	N_8_	0.055	0.059
N_6_	0.033	0.039	N_5_	0.033	0.039	C_10_	0.041	0.051
C_8_	0.074	0.036	C_7_	0.071	0.037	N_5_	0.025	0.036
C_7_	0.057	0.023	N_13_	0.051	0.023	C_7_	0.049	0.033
N_14_	0.052	0.023	C_6_	0.054	0.023	N_13_	0.042	0.022
C_1_	0.015	0.019	C_1_	0.013	0.017	C_6_	0.034	0.021
C_2_	0.013	0.013	C_2_	0.008	0.011	C_1_	0.01	0.016
C_3_	0.007	0.009	C_25_	0.01	0.008	C_2_	0.007	0.014
O_4_	0.007	0.005	C_29_	0.004	0.002	C_3_	0.013	0.006
—	—	—	C_3_	0.002	0.001	—	—	—

a Calculated using Hirshfeld charges at B3LYP/6-31G(d,p).

#### 3.5.4. Monte Carlo simulations

**Fig. 16:**
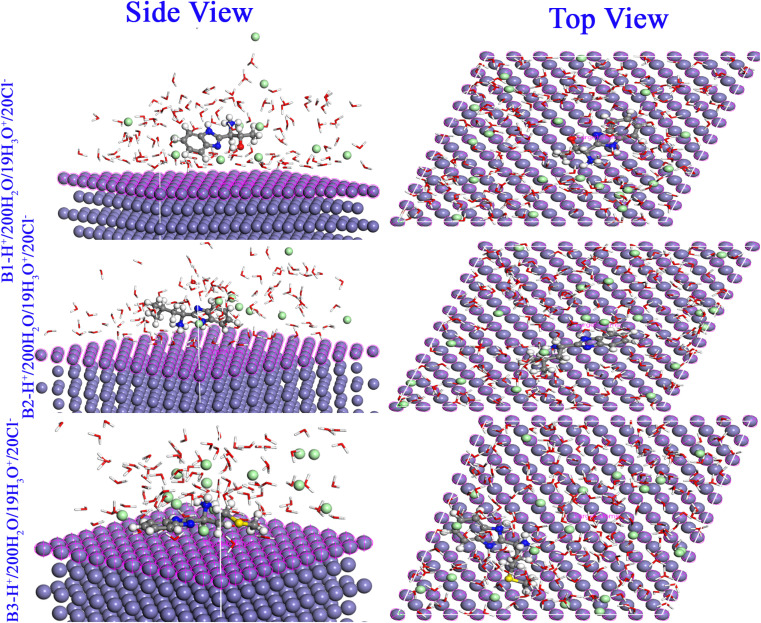
Monte Carlo simulations for the most favorable modes of adsorption obtained for B1-H^+^, B2-H^+^ and B3-H^+^ on Fe (1 1 0) surface, side and top view.

The locations and adsorption arrangements of the B1, B2 and B3 molecules investigated in the Fe substrate are shown in Fig. 16 and the different adsorption energies values are displayed in Table 10. From Fig. 16, it is obvious that the compounds B1, B2 and B3 adsorb to the surface of Fe (110) with a nearly flat orientation. In accordance with the FMO, NBO and Fukui data, the heteroatoms (oxygen, sulfur, and nitrogen) lone pairs and π electrons either conjugated or isolated can transfer to the empty orbitals of the metallic substrate *via* strong coordination links.^85^ The active sites distribution over the whole molecular skeleton of B1, B2 and B3 molecules guarantee maximum size adsorption and strong adherent protection layer formation that protect against corrosion.^86^ At different modes of calculations (gas or aqueous), B1, B2 and B3 molecules obtained high negative values of adsorption energies to reveal a spontaneous favored adsorption. The values of binding energies enhanced for the protonated form to approach 380.794 kcal mol^−1^ for B3 molecule. The binding energies of B1, B2 and B3 molecules are greater than those of H_2_O molecules (in our calculation binding energies of water molecules ranged from 6.599 to 11.368), which supports the progressive replacement of H_2_O molecules from the surface of X56 c-steel.^87^ These energy properties support the concept that the prevention of X56 c-steel corrosion was accomplished by the adsorption of the investigated molecules by the order B3 > B2 > B1.

**Table 10:** The outputs and descriptors calculated by the Monte Carlo simulations for adsorption of B1, B2 and B3 on Fe (110) (in kcal/mol)

Phase	Inhibitor	Total energy (kcal mol^−1^)	Adsorption energy (kcal mol^−1^)	Rigid adsorption energy (kcal mol^−1^)	Deformation energy (kcal mol^−1^)	(d*E*_ads/dNi_) (kcal mol^−1^)	Binding energy (kcal mol^−1^)	IE^a^ (%)
Gas phase	B1	−10.912	−249.383	−106.185	−143.198	−249.383	249.383	92.11
	B2	−41.150	−254.592	−122.197	−132.395	−254.592	254.592	94.64
	B3	−38.797	−254.654	−117.706	−136.947	−254.654	254.654	95.92
	B1–H^+^	−19.852	−334.489	−102.445	−232.043	−334.489	334.489	92.11
	B2–H^+^	−19.222	−347.288	−114.273	−233.015	−347.288	347.288	94.64
	B3–H^+^	−23.153	−347.402	−120.144	−227.258	−347.402	347.402	95.92
Aqueous phase	B1	−5903.928	−6301.869	−6147.089	−154.779	−165.213	165.213	92.11
	B2	−5981.094	−6354.005	6211.268	−142.737	−222.146	222.146	94.64
	B3	−5967.283	−6342.608	6188.060	−154.547	−266.108	266.108	95.92
	B1–H^+^	−5787.303	−6280.860	−6019.430	−261.430	−248.532	248.532	92.11
	B2–H^+^	−5855.750	−6375.581	−6125.079	−250.50	−254.802	254.802	94.64
	B3–H^+^	−5904.997	−6394.736	−6149.752	−244.984	−380.794	380.794	95.92

a Inhibition efficiency values obtained from EFM measurements.

## 4. Conclusions

Organic synthesis was conducted to obtain three new corrosion inhibitors to response to the vital and continues demand of carbon steel (X56) protection to alleviate the pressure on the petroleum industry as a result of the losses caused by metal corrosion. The synthesis included amino acids benzimidazole base derivatives that are expected to be a strong adsorbate to metal surface and at the same time cheap adhering films. The conjugated system in the benzimidazole moieties besides the lone pairs of heteroatoms and the donating power of the methyl groups facilitate the adsorption to the metallic surface with priority of B3 due to the high donating power of S atom. The blocking of corrosion sites was confirmed by EFM, EIS and PDP electrochemical measurements. In addition, the EFM data were fitted to six adsorption isotherms to validate the attachment to the metal surface. An overall inhibition efficiency of average 95–98% was obtained for both anodic and cathodic corrosion reactions through Langmuir adsorption of the inhibitor to X56 surface. SEM, in addition to FESEM, revealed an effective surface protection for inhibitor solutions rather than the blank one. DFT calculations confirmed a feasible and strong electron reallocation from the inhibitor HOMO to the metal surface.

## Conflicts of interest

The authors declare that they have no known competing financial interests or personal relationships that could have appeared to influence the work reported in this paper.

## Supplementary Material
